# Supplementary material for: All-optical strategies to minimize photobleaching in reversibly switchable fluorescent proteins
Source: Nat Commun. 2025 Dec 1;16:10843. doi: 10.1038/s41467-025-67009-8 (PMC12672575; doi:10.1038/s41467-025-67009-8)
Supplement: Supplementary file 1 — Supplementary Information [file 41467_2025_67009_MOESM1_ESM.pdf]

Supplementary Information for

All-optical strategies to minimize photobleaching in reversibly switchable fluorescent proteins

Guillem Marín-Aguilera, Francesca Pennacchietti, Andrea Volpato, Alessia Papalini, Abhilash Kulkarni, Niusha Bagheri, Guillaume Minet, Jerker Widengren and Ilaria Testa.

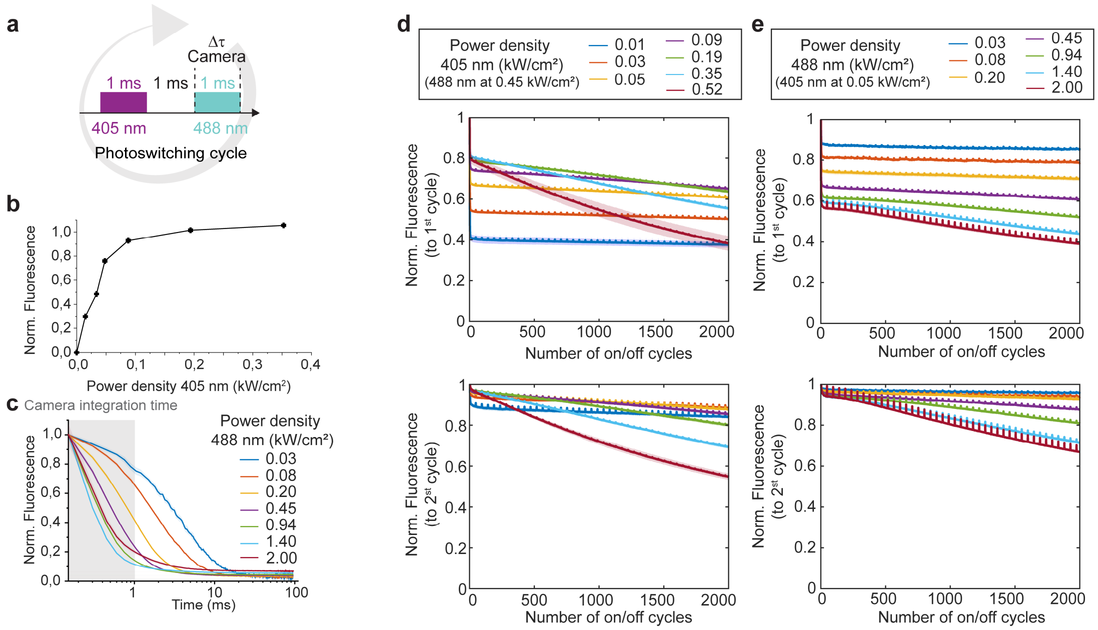

Supplementary Figure 1. Photoswitching fatigue characterization. (a) Schematic of the photoswitching fatigue experiment. rsEGFP2-embedded PAA gel is repeatedly illuminated with 1 ms 405 nm and 1 ms of 488 nm light, with an interval of 1 ms between them. An interval of two orders of magnitude in power density is explored for both wavelengths, from ~ 0.01 to ~ 1 kW/cm^2. (b) Moving the 405 nm illumination across this range results in the complete on-switch of the RSFPs fraction, (c) while the increased 488 nm speed up the off-switch kinetics. The grey area in the graph corresponds to the illumination time and consequently camera integration time used for the photoswitching fatigue recording. The set time of 1 ms matches the 80 % decrease in fluorescence for the middle power density. Photoswitching fatigue recorded at varying (d) 405 nm and (e) 488 nm (mean ± σ of at least 3 measurements). The upper graphs are normalized to the fluorescence of the first cycle, while the bottom one is normalized to the second cycle to better visualize and compare the fatigue fraction upon the different illuminations.

Supplementary Note 1: Modelling of rsEGFP2's photocycle

The mechanism that drives the reversible photoswitching in rsEGFP2 consists of an excited state photoisomerization reaction followed by a protonation/deprotonation process[1–5]. Supplementary Figure 2a shows the 4 main "fully relaxed" states involved in the photoswitching, the neutral and anionic *cis* (*CH*, C^{-}) and neutral and anionic *trans* (*TH*, T^{-}) chromophore species. The protonation state of the chromophore defines the spectral properties of a given electronic state[4], in that sense, deprotonated states (C^{-} and T^{-}) primarily absorb blue light while protonated states (*CH* and *TH*) have their absorption

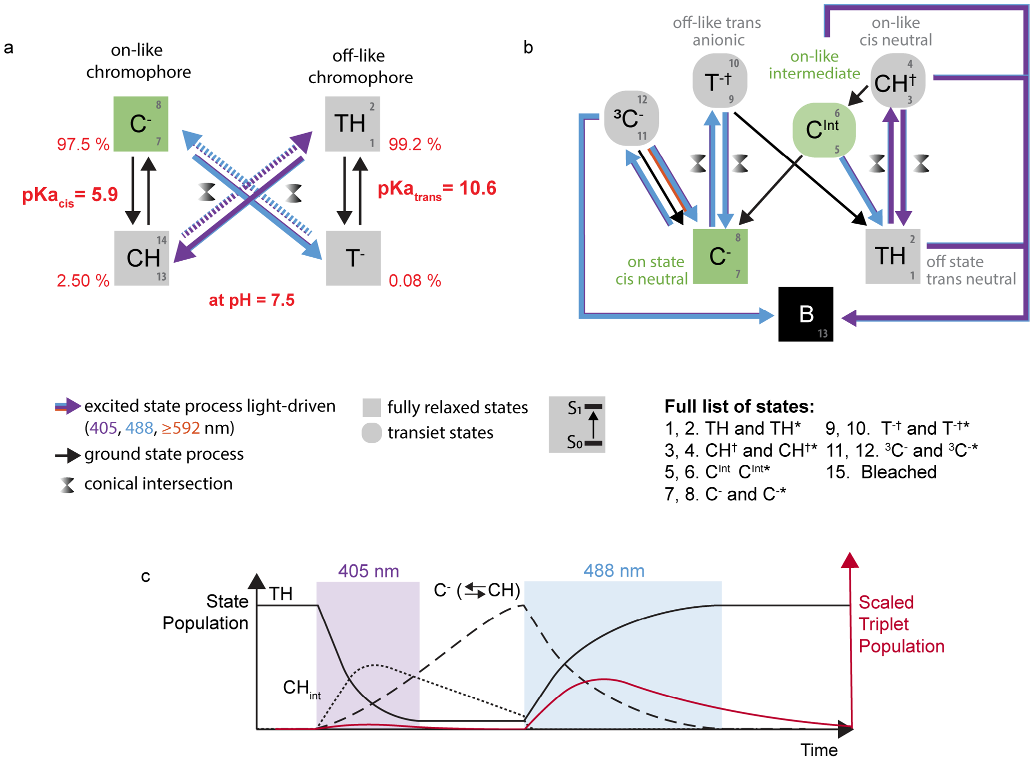

Supplementary Figure 2. (a) "Fully relaxed" states involved in the photocycle of rsEGFP2. The on-state C^{-} is in pH equilibrium with *CH*, a state we named Cis-on-like neutral. At physiological pH, the predominant form is C^{-}. On the other hand, the off-state *TH* is in pH equilibrium with T^{-}, a state we named Trans-off-like anionic. Species with the same protonation state can interconvert into each other via light-induced photoisomerization. The solid arrows indicate the more relevant pathways in the photoswitching of the protein. (b) Kinetic scheme for rsEGFP2 used in simulations. The scheme above accounts for the excited state isomerization reactions that drive the photoswitching, the presence of the intermediate state (C^{Int}) in the on-switching process, the protonation/deprotonation processes, the formation of the triplet state from the fluorescent form ($^{3}C^{-}$) and the different effective bleaching channels. The cyan, violet, and orange arrows aim to represent the wavelength that drives the transition between the different electronic states. The green-shaded states represent those with fluorescence emission, both from the C^{Int} and the C^{-} forms. The "fully relaxed" states are represented at the bottom and shaped like squares. Grey arrows describe processes from the excited state: non-radiative relaxations or excited state reactions. Black arrows depict transitions that occur in the ground state. As presented, the proposed kinetic scheme aims to reproduce three main phenomena: the photoswitching of rsEGFP2 (on-to-off and off-to-on transitions); the formation of triplet state via intersystem crossing and the triplet's interaction with light; and lastly, the observed loss of signal due to photobleaching. List of states: 1-Trans neutral; 2-Trans neutral excited; 3-Cis-off-like neutral; 4-Cis-off-like neutral excited; 5-Intermediate, 6-Intermediate excited; 7-Cis anionic (ON); 8-Cis anionic (ON) excited; 9-Trans-on-like anionic; 10-Trans-on-like anionic excited; 11-Triplet; 12-Triplet excited; 13-Bleached. (c) Representation of the time evolution of TH, C^{Int} and C^{-} over the sequential illumination profile generally used in the experiment, i.e. 488 and 405 nm interleaved with a delay without any illumination.

maximum around 400 nm. From the *cis* conformation only the deprotonated state C^- is fluorescent[1–3] and at pH = 7.5[6] is the favoured "on-like" state ($[C^-]/[CH]$ ~ 40). The *trans* isomers are the "off-like" states with *TH* being favoured at pH=7.5 ($[TH]/[T^-]$ ~ 1200). Although photoisomerization can proceed from both protonated and deprotonated chromophore, Given the distribution of concentrations at pH=7.5, we deemed the photoswitching reactions stemming from C^- (off-switching) and *TH* (on-switching) as more relevant to be explored in this work (solid line in Supp. Fig. 1a).

To incorporate bleaching in the photoswitching of rsEGFP2 it is necessary to expand the simple kinetic model incorporating different excited state processes and states. Supplementary Figure 2b reports all the photophysical pathways included in our model.

Among the previously "fully relaxed" states we consider only the main populated ones at our working pH of 7.4: C^- (on-state) and *TH* (off-state). The bleached state (*B*) is also considered as "fully relaxed" since there are no further reactions that can stem from it. From *TH* the on-switching transition occurs as an excited state isomerization reaction via a conical intersection to an "off-like" neutral chromophore transient state, $CH^{\dagger}$, analogous to the *Cis on-like neutral chromophore* form characterized in Uriarte et al.[2] that will rapidly relax (~ µs[1–3]) to form another transient state we named C^{Int}. Our data suggest this intermediate to be emissive, with a relaxation time of ~ 1 ms to C^- (Supplementary Note 3). Once C^- is reached, a rapid proton exchange equilibrium (35 µs[1]) establishes between the C^- and the *CH* form. We did not incorporate *CH* in the scheme given that at pH = 7.4, $[C^-]/[CH]$ ~ 40 and 405 nm light is illuminated on the sample mainly on the off-state (Supplementary Figure 2c). The on-switching is mainly triggered at near UV illumination (commonly 405 nm), while the off-switching transition occurs upon illumination with blue light (commonly 488 nm). 488 nm illumination triggers an excited state isomerization reaction via a conical intersection from C^- to T^-, the "on-like" trans anionic form of the chromophore. Such as for the on-like form, we considered $T^{-\dagger}$, transient state that rapidly (~ 50 µs[5]) protonates to form the off-state (*TH*) at pH 7.4.

Our kinetic model also includes the formation of a triplet state ($^3C^-$) from the on-state C^- via intersystem crossing (ISC). The triplet state will decay non-radiatively to the on-state with a lifetime of around ~ 3 ms (Supplementary Note 7). Given the broad triplet state absorption spectrum of rsEGFP2[7], we acknowledge that $^3C^-$ can undergo reverse intersystem crossing (RISC) and repopulate C^-. As established in EGFP[8], the triplet state is the gateway for irreversible photobleaching reactions in fluorescent proteins. We have included such pathways from both the ground and excited triplet states. To reproduce the dataset of Figure 1d, we have included bleaching pathways from the "fully relaxed" off-state *TH*.

The assumptions made in building up the photoswitching model are the following.

- Triplet formation via ISC only appears from the C^-, although the C^{Int} state is also described as fluorescent. We reason that since in all characterization experiments there exists a delay of 1 ms between the 405 and 488 nm illumination, most of the C^{Int} will be converted into C^- thus not contributing significantly to the overall triplet formation (Supplementary Figure 2c).
- Isomerization from C^{Int} to *TH* as a one-step excited state reaction. Although it is plausible that a cascade of intermediary states with characteristic lifetimes guides the isomerization reaction from C^{Int} to *TH*, the timescale of our experimental setting is too slow to distinguish among them, therefore we have considered the reaction as one only step.

- Any absorption from $^{3}C^{-}$ will lead to the same "effective" excited triplet state ($^{3}C^{-}$*) and that RISC will repopulate the excited state of the emissive form (C^{-}*). Considering the full complexity of the triplet state, RISC could happen from T_n to a highly energetic vibrational state of S_0 or S_1.[10] Nevertheless, our experimental data, where we probe slow processes in the ms-to-s time scale, are unable to distinguish between the two pathways. With an effective quantum yield Φ_{RISC} both pathways can reproduce the described light-induced recovery. To build a minimal effective kinetic model and following the triplet absorption spectrum reported for rsEGFP2[11,12] we have described RISC from T_n effective triplet excited state to S_1. In support of this assumption are the optically-activated delayed fluorescence (OADF)[13,14] reported in fluorescent proteins, where RISC leads to repopulation of the singlet excited state of the emissive form.

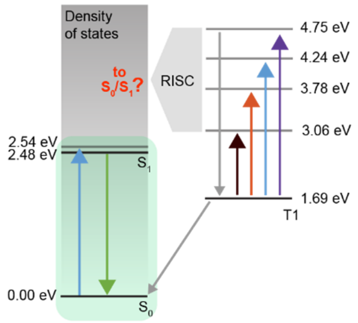

Supplementary Figure 3. Schematic of energy ladder of singlet and triplet states for rsEGFP2 in eV. The position of S_1 was determined from the maximum in the emission spectra[9]. The position of T_1 was determined from the maximum in phosphorescence emission spectra of EGFP[8] as an approximation of the energy level of the first triplet state of rsEGFP2. The energy levels of the subsequent excited triplet states were calculated as $E(\lambda) = 1.69 + 1.24/\lambda$, with λ in µm. The coloured arrows indicate which wavelength would populate each of the energy levels: 3.06 eV, 900nm; 3.78 eV, 592 nm; 4.24 eV, 488 nm; 4.75 eV, 405 nm. Line arrows indicate the different RISC mechanisms that were simulated.

In parametrizing the outlined model, electronic states with the same protonation state have been considered to share the same spectroscopic properties, that is the extinction molar coefficients, $\varepsilon^{TH}_{\lambda} \approx \varepsilon^{CH\dagger}_{\lambda}$ and $\varepsilon^{C-}_{\lambda} \approx \varepsilon^{T-\dagger}_{\lambda}$ as well as the quantum yields for the *cis-trans* isomerization $\Phi_{TH\rightarrow CH\dagger} \approx \Phi_{CH\dagger\rightarrow TH}$ and $\Phi_{C-\rightarrow T^{\dagger}-} \approx \Phi_{T^{\dagger}-\rightarrow C}$. These parameters have been extracted from the literature and properly referenced in Supplementary Table 2. From Supplementary Table 1, $\sigma(\lambda)$ refers to the absorption cross-section for a given wavelength in each state. $\sigma(\lambda)$ is calculated from $\varepsilon(\lambda)$ as indicated in literature[15]. $\rho(\lambda)$ is the photon flux delivered to the sample as calculated from the power density measured at the objective's back aperture and the size and shape of the beam profile. Φ refers to the quantum yield of different processes and τ are the lifetimes of the excited states. The different transitions between states are listed in Supplementary Table 1 as i.e. K_{21}, which indicates a process that moves population *to* state *2 from* state *1*. All the parameters used in the simulations are listed and referenced below in Supplementary Table 2.

The purpose of the simulation was to parametrize and help clarify the photophysical pathways that could describe the experimental observations; therefore, not all the simulated experiments required the same level of complexity from the kinetic scheme. A reduced kinetic model including only electronic states involved in the photoswitching (C^{-}, $CH^{\dagger}$, C^{Int}, TH and $T^{\dagger}$) were used to estimate the absorption parameters of the C^{Int} as discussed in Supplementary Note 3. Since such experiments did not require many photoswitching cycles, the formation of a $^{3}C^{-}$ nor the different bleaching pathways were included in the modelling of rsEGFP2. On the other hand, the full kinetic scheme of Supplementary Figure 2b

was required to estimate the absorption properties of the triplet state as well as when discussing the different bleaching pathways that intervene in the destruction of the fluorescence signal upon cycling.

Supplementary Table 1. Index of the transitions between electronic states of Supplementary Figure 2b. The different transitions are described as i.e. K_{21}, which indicates a process that moves the population *to state 2 from state 1*.

K	Equation	Description
**K_{21}**	$\sigma_{TH}(\lambda) * \rho(\lambda)$	Trans neutral absorption
**K_{12}**	$1/\tau_{TH}$	Trans neutral relaxation
**K_{32}**	$\Phi_{TH \rightarrow CH\dagger} / \tau_{TH}$	Trans to cis-off-like-transient isomerization
**K_{43}**	$\sigma_{CH\dagger}(\lambda) * \rho(\lambda)$	Cis-off-like absorption
**K_{34}**	$1/\tau_{CH\dagger}$	Trans neutral relaxation
**K_{14}**	$\Phi_{CH\dagger \rightarrow TH} / \tau_{CH\dagger}$	Cis-off-like to trans neutral isomerization
**K_{53}**	$1/\tau_{Rearrangement}$	Reorganization of the chromophore in the pocket
**K_{65}**	$\sigma_{Cint}(\lambda) * \rho(\lambda)$	Intermediate absorption
**K_{56}**	$\Phi_{fluo} / \tau_{ICint}$	Fluorescence emission
**K_{16}**	$\Phi_{Cint \rightarrow TH} / \tau_{Cint}$	Intermediate-to-trans-neutral isomerization
**K_{87}**	$\sigma_{C-}(\lambda) * \rho(\lambda)$	Cis-on anionic absorption
**K_{78}**	Φ_{fluo} / τ_{C-}	Fluorescence emission
**K_{98}**	$\Phi_{C- \rightarrow T-\dagger} / \tau_{C-}$	Cis to trans-on-like anionic isomerization
**K_{109}**	$\sigma_{T-\dagger}(\lambda) * \rho(\lambda)$	Trans-on-like anionic absorption
**K_{910}**	$1/\tau_{T-\dagger}$	Trans-on-like anionic relaxation
**K_{710}**	$\Phi_{T-\dagger \rightarrow C-} / \tau_{T-\dagger}$	Trans-on-like to cis anionic isomerization
**K_{19}**	$1/\tau_{protonation\text{-}T\text{-}\dagger}$	Protonation of the chromophore in the trans-on-like isomer
**K_{118}**	Φ_{ISC} / τ_{C-}	Triplet state formation via intersystem crossing
**K_{711}**	$1/\tau_{Triplet}$	Triplet relaxation
**K_{1211}**	$\sigma_{Triplet}(\lambda) * \rho(\lambda)$	Triplet absorption
**K_{1112}**	$1/\tau_{Triplet\text{-}Excited}$	Triplet excited relaxation
**K_{812}**	$\Phi_{RISC} / \tau_{Triplet\text{-}Excited}$	Reverse intersystem crossing to the single state
**K_{132}**	$K_{bleach\text{-}Off\text{-}to\text{-}On}$	Bleaching from the trans neutral
**K_{134}**	$K_{bleach\text{-}Off\text{-}to\text{-}On}$	Bleaching from the cis-off-like neutral
**K_{136}**	$K_{bleach\text{-}Off\text{-}to\text{-}On}$	Bleaching from the cis intermediate
**K_{1311}**	$\Phi_{Bleach\text{-}triplet} / \tau_{Triplet}$	Bleaching from the triplet
**K_{1312}**	$\Phi_{Bleach\text{-}triplet\text{-}excited} / \tau_{Triplet\text{-}Excited}$	Bleaching from the triplet excited

Supplementary Table 2. List of parameters used in the simulations.

Parameter	Value	Reference
$\varepsilon^{TH}{}_{405} = \varepsilon^{CH\dagger}{}_{405} = \varepsilon^{OFF}{}_{405}$	22000 M^{-1} cm^{-1}	[6]
$\varepsilon^{TH}{}_{488} = \varepsilon^{CH\dagger}{}_{488} = \varepsilon^{OFF}{}_{488}$	60 M^{-1} cm^{-1}	[6]
$\varepsilon^{T\text{-}\dagger}{}_{405} = \varepsilon^{C\text{-}\dagger}{}_{405} = \varepsilon^{ON}{}_{405}$	5260 M^{-1} cm^{-1}	[6]
$\varepsilon^{T\text{-}\dagger}{}_{488} = \varepsilon^{C\text{-}}{}_{488} = \varepsilon^{ON}{}_{488}$	61560 M^{-1} cm^{-1}	[6]
$\varepsilon^{Int}{}_{405}$	16555 M^{-1} cm^{-1}	Data
$\varepsilon^{Int}{}_{488}$	28000 M^{-1} cm^{-1}	Data
$\tau_{TH} = \tau_{CH\dagger} = \tau_{OFF}$	20 ps	[1]
$\tau_{T\text{-}\dagger} = \tau_{C\text{-}} = \tau_{Int} = \tau_{ON}$	1.6 ns	[16]
$\tau_{Rearrangement}$	5.1 µs	[1]
$\tau_{Relaxation\text{-}Cint}$	825 µs	Data
$\tau_{protonation\text{-}T\text{-}\dagger}$	48 µs	[5]
Φ_{fluo}	35 %	[6]
$\Phi_{CH\dagger\rightarrow TH} = \Phi_{TH\rightarrow CH\dagger}$	33 %	[6]
$\Phi_{T\text{-}\dagger\rightarrow C\text{-}} = \Phi_{C\text{-}\rightarrow T\text{-}\dagger}$	1.7 % *	[6]
$\Phi_{Int\rightarrow TH}$	12.6 %	Data
pK_a	5.9	[6]
$\tau_{Triplet}$	5 ms	[7]
$\tau_{Triplet\text{-}Excited}$	1.0 ps	[17]
Φ_{ISC}	0.25 %	[7]
Φ_{RISC}	0.25 %	Data
$\varepsilon^{Triplet}{}_{405}$	2000 M^{-1} cm^{-1}	[7]
$\varepsilon^{Triplet}{}_{488}$	10000 M^{-1} cm^{-1}	[7]
$\varepsilon^{Triplet}{}_{592}$	7500 M^{-1} cm^{-1}	[7]
$K_{Bleach\text{-}Off\text{-}to\text{-}On}$	~ 10^{-1} – 10^{0} ms^{-1}	Data
$\Phi_{Bleach\text{-}triplet}$	~ 10^{-3}	Data
$\Phi_{Bleach\text{-}triplet\text{-}excited}$	~ 10^{-6} – 10^{-7}	Data
$\varepsilon \rightarrow \sigma$ (conversion factor)	3.825 x 10^{-21} M cm^{-1}	[15]

* Updated value for $\Phi_{T\text{-}\dagger\rightarrow C\text{-}} = \Phi_{C\text{-}\rightarrow T\text{-}\dagger}$ in [18].

Supplementary Note 2: Origin of the initial drop in rsEGFP2 photoswitching fatigue curve

The photocycle of rsEGFP2 has been exhaustively studied using different spectroscopic techniques[1–3,5] as well as crystallographic methods[6,9]. In particular, the off-to-on transition has been characterized as a multi-step process involving an excited state isomerization reaction followed by the deprotonation of the chromophore[1–3]. The final step results from a cascade of intermediates with different lifetimes ranging from 5 µs to 2000 µs[1,2]. At moderate 405 nm energy doses (typically for RESOLFT microscopy ~ 10 – 500 W/cm^2), the lifetimes of the different intermediates become the rate-limiting step of the on-switching process. Moreover, since the isomerization process is rather efficient ($\boldsymbol{\Phi}_{TH\rightarrow CH^{\dagger}}$ = 0.33[6]), an excess of UV illumination will lead to a light-induced quasi-equilibrium between the different protonated isomers of the chromophore.

We reason that the presence of a long-lived C^{Int} and a large absorption cross-section at 405 nm is behind the large initial drop in the recorded fluorescence signal on the photoswitching fatigue experiments. To illustrate the effect, we simulated the expected fluorescence response of rsEGFP2 to consecutive photoswitching cycles, just as it occurs in a fatigue experiment, and monitored the drop in the fluorescence signal from the 1st to the 2nd cycle for different C^{Int} lifetimes in a 5-state model.

As the 405 nm on-switching dose is applied, the stable off-state *TH* is rapidly converted into $CH^{\dagger}$ and further on to C^{Int} from where it can reach the on-state, C^{-} via a slow ground-state relaxation. Nonetheless, the intermediate state can also undergo photoisomerization back to *TH* by the same 405 nm dose reducing the final on-state concentration.

In general, if both on and off-states have an absorption cross-section for both on/off-switching wavelengths the cycling between the two states will never be 100%[19]. The presence of an intermediate state in the *off-to-on* transition adds another pathway that can further reduce the efficiency of the on-switching process since this state can also be excited by on/off-switching wavelengths. As shown in

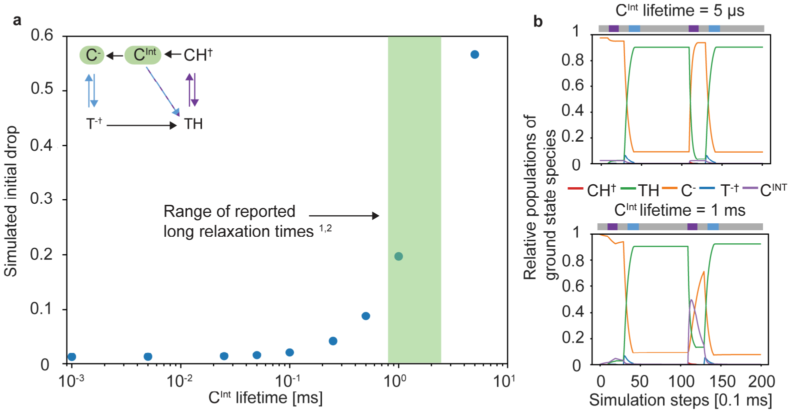

Supplementary Figure 4. (a) Simulated drop of fluorescence between the 1st and 2nd cycles in a fatigue experiment as a function of the C^{Int} lifetime. The inset shows a schematic of the proposed photoswitching model for rsEGFP2 where C^{-} is the fluorescent state or on-state, and *TH* is the main off-state. As the C^{Int} lifetime increases, the probability of a UV-light-triggered isomerization reaction from the C^{Int} to the off-state also increases, reducing the fluorescence observed in the second cycle. The green-shaded area shows the range of the longest relaxation times in the literature[1,2]. The illumination doses set as input in the simulations are comparable to those of an experimental setting: 405 nm exposure for 1 ms at 100 W/cm^2 and 488 nm exposure for 1.2 ms at 200 W/cm^2. (b) Time traces of the relative populations of the ground state species involved in the photoswitching of rsEGFP2. For short C^{Int} lifetimes, there is no accumulation of the C^{Int} population, and the majority of the *TH* concentration can be on-switched to C^{-}. On the other hand, longer C^{Int} lifetimes, result in a residual fraction of the protein's ensemble population being trapped in *TH* after on-switching.

Supplementary Figure 4a, the initial drop is non-zero for any C^{Int} lifetimes and becomes more relevant around τ ~ 1 ms at 405 nm power density = 100 W/cm^2 and 1 ms of illumination time. By examining the time traces of the relative populations of the ground state species involved in photoswitching, we observe that a longer C^{Int} lifetime reduces the concentration of molecules in the on-state at the second photoswitching cycle (orange curves in Supplementary Figure 4b), thus, yielding a lower fluorescence signal. Additionally, for a C^{Int} lifetime of 1 ms the C^{Int} species will accumulate at the onset of 405 nm illumination (purple curve) forming a quasi-equilibrium between C^{Int} , $CH^{\dagger}$ and *TH* which equilibrates around 20% of the population in the off-state, *TH* (green curve in Supplementary Figure 4b).

Supplementary Note 3: Parametrization of the intermediate species of rsEGFP2

To model the photoswitching cycle of rsEGFP2 most reliably, some of the parameters concerning the C^{Int} species had to be elucidated, in particular, the absorption properties at 405 and 488 nm ($\varepsilon^{Int}{}_{405}$ and $\varepsilon^{Int}{}_{488}$, respectively) as well as the $\Phi_{Int\rightarrow TH}$.

To parametrize the extinction coefficient at 488 nm, the on-switching process of rsEGFP2 was characterized with µs temporal precision using the confocal microscope described in Supplementary Note 15 and the rsEGFP2 protein embedded in a polyacrylamide (PAA) gel. The fluorophore population residing in a diffraction-limited confocal volume was firstly off-switched with a long 488 nm light dose (3 ms and ~ 50 kW/cm^2). Given that there is a non-zero probability of on-switching at 488 nm, there will be a residual signal at the end of that illumination pulse and this background level is subtracted from the signal of the overall time trace. Immediately after, a burst of 405 nm light (5 µs and ~ 40 kW/cm^2) was delivered in the same volume to trigger the on-switching transition. Following a variable delay (5 µs up to 51.2 ms), another 488 nm dose (0.5 ms and ~ 50 kW/cm^2) was delivered to the sample, and the evolution of the fluorescence signal was recorded. As shown in Supplementary Figure 5a, the immediate fluorescence response gradually increases with the delay between 405 and 488 nm pulses, reaching a plateau after a few ms. Additionally, we observed that for short delays – 5 and 10 µs – there is a build-up of the fluorescence signal in the first few microseconds of 488 nm illumination (Supplementary Figure 4b) compatible with the first relaxation step in the on-switching transition[1,2]

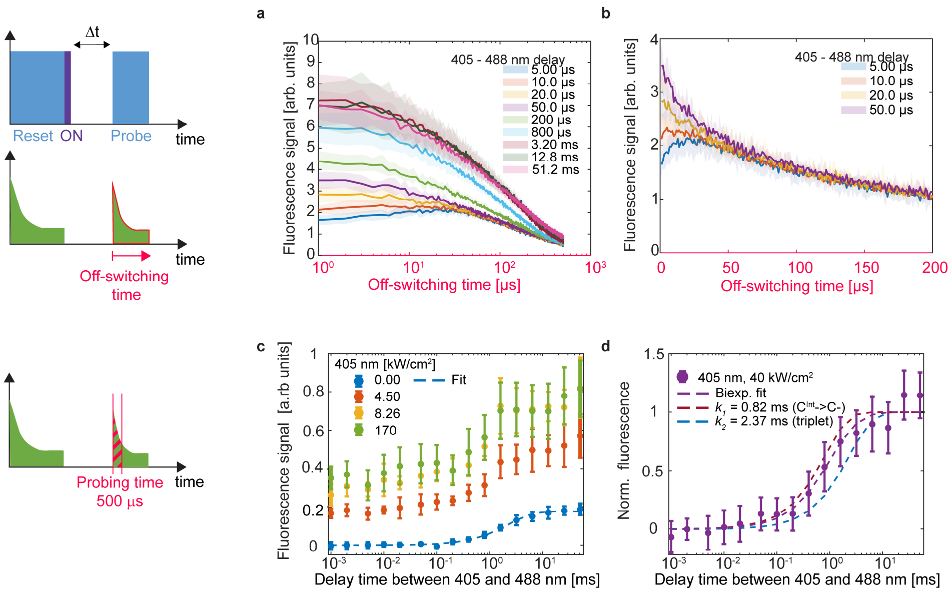

Supplementary Figure 5. (a) Off-switching curves for multiple 405-488 delays. Longer delay between pulses correlated with an increase of the fluorescence signal peak up to a few ms when the maximum fluorescence value reached a plateau. (b) Initial 200 µs of off-switching curves for short delays (5 µs to 50 µs). The data show there is a build-up of the signal in the tenths of microseconds timescale after 488 nm illumination for short delays (5 and 10 µs). (c) Integrated fluorescence signal as a function of the delay between 405 and 488 nm pulses at different 405 nm power densities. Even with no 405 nm illumination (blue line), there is a raise in signal with a characteristic time $t_2 = 2.37 \pm 0.40$ ms, that we assign to the triplet state. The data is fitted with a raising monoexponential function, $y_2 = a_2(1\text{-}exp(\text{-}t/t_2))$. As the 405 nm illumination is introduced, the signal at shorter delays increases and a faster component also appears in the raising signal. (d) Normalized fluorescence captured as function of delay between 405 and 488 nm pulses at a 405 nm power density of 40 kW/cm^2. For visualization purposes, the data and corresponding fits have been normalized between 0 and 1. A biexponential function was necessary to fit the data. The slower component in blue, corresponds to the relaxation of the triplet state, while the faster component we attribute to C^{Int}. The characteristic time of the faster component is $t_1 = 0.82 \pm 0.45$ ms, close to the slow relaxation processes observed in TA measurements[1,2].

($CH^{\dagger} \rightarrow C^{Int}$ in our scheme of Supplementary Figure 2b) and earlier reported in time-resolved fluorescence decays in the µs time range[5].

We pinpoint the C^{Int} species to be responsible for the fluorescence signal recorded after a 1 µs delay, which is around 40 % of the maximum value. Given the reported extinction coefficient of the fluorescent form, $\varepsilon^{On}{}_{488}$ = 61900 M^{-1} cm^{-1} [6], our data suggest that $\varepsilon^{Int}{}_{488}$ ~ 28000 M^{-1} cm^{-1}. A key assumption was that C^{Int} and C^{-}, share fluorescence lifetime and fluorescence quantum yield, but differ in their cross-sections at 405 and 488 nm. This is a simplification that we made in the absence of better knowledge of the state C^{Int}. Nevertheless, this choice is guided by recent ultrafast spectroscopical data[1,2]. Since the timescales of the explored delay are in the range of the triplet relaxation, we have performed a control experiment at no 405 nm to calibrate the influence of the triplet relaxation in the observed dynamic. We fit the observed relaxation with no 405 nm (Supplementary Figure 4c, blue line) with a monoexponentially increasing function yielding t_2 = 2.37 ± 0.40 ms and an amplitude of a_2 = 0.175 ± 0.081. To elucidate the time constant of the fluorescence signal raise of Supplementary Figure 5d, we employ two exponentials, one coming from the triplet state raise, and we fit the other component which returns a faster raise with t_1 = 0.82 ± 0.45 ms and an amplitude of a_1 = 0.374 ± 0.057. As shown in Supplementary Figure 5d, the signal increase with respect to the delay between pulses is well captured by the biexponential fit, showcasing a faster time evolution than that of the triplet phase. The timing of the faster component in the biexponential function matches well the slower relaxation times observed in transient absorption measurements of rsEGFP2[1,2]. For visualization purposes, the data and the corresponding fits in Supplementary Figure 5d have been normalized between 0 and 1. The parameters from the fit are summarized in Supplementary Table 3.

On the other hand, the $\varepsilon^{Int}{}_{405}$ and $\Phi_{Int\rightarrow TH}$ were estimated by fitting the photoswitching model of rsEGFP2 (electronic states 1-10 from Supplementary Figure 2b) to a 405 nm on-switching ramp of increasing activation doses. The experiment consisted of consecutive photocycles, 405 nm to on-switch and 488 nm to off-switch and fluorescence read-out, with variable 405 nm power densities (up to 0.6 kW/cm^2) to elucidate the fluorescence signal dependence to the on-switching dose. We decided to fix the C^{Int} lifetime during the fitting routine to the slowest relaxation process observed by TA spectroscopy[1].

As shown in Supplementary Figure 6, the observed signal increases rapidly with increasing 405 nm illumination until it reaches a plateau (around 100 W/cm^2), where the signal saturates. The same experiment was simulated with a 5-state photoswitching model (electronic states indexed 1-10 in Supplementary Figure 2b) of rsEGFP2. No triplet state formation nor bleaching pathways were considered as each data point was an average of 9 photocycles and our photoswitching fatigue data do not show substantial bleaching effects after so few cycles. The output of the simulation was a modelled on-switching curve that was passed to a least-square fitting routine with $\varepsilon^{Int}{}_{405}$ and $\Phi_{Int\rightarrow TH}$ as the parameters to estimate. The best fit was found at $\varepsilon^{Int}{}_{405}$ ~ 16000 $M^{-1}cm^{-1}$ and $\Phi_{Int\rightarrow TH}$ ~ 12%. To our knowledge, there are no studies parametrizing the spectroscopic properties of C^{Int} and the values we

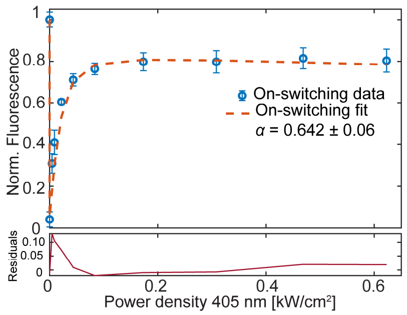

Supplementary Figure 6. On-switching curve data and fit. Each data point is an average of 9 complete photocycle repetitions. The simulation consisted of a 5-state kinetic model without triplet and bleaching pathways.

obtained from the fit are estimations given the assumed photoswitching model. However, the evolution of the UV-vis spectra of rsEGFP2 has been characterised by time-resolved transient absorption spectroscopy data[1], it points to a gradual decrease in the band around 405 nm indicating that the absorption in the UV region lowers as the chromophore changes from the off-state (ε^{Off}_{405} = 22000 M^{-1}cm^{-1} [6]) to the on-state (ε^{On}_{405} = 5260 M^{-1}cm^{-1} [6]). This behaviour is consistent with the value obtained in the fit.

Supplementary Table 3. Estimated parameters for the C^{Int} state. The value of the parameters is accompanied by the confidence interval calculated from their respective fitting routines.

Parameter		Method	Estimated value	Confidence Interval (95%)
ε^{Int}_{488}		Time-resolved fluorescence measurement	28000 M^{-1} cm^{-1}	-
$y_2 = a_2\,(1\text{-}exp(\text{-}t/t_2))$	a_2	Monoexponential fit	0.175	0.167 – 0.183
	t_2		2.37 ms	1.97 – 2.77 ms
$y_{bi} = b - a_1{*}exp(\text{-}t/t_1) + y_2$	a_1	Biexponential fit	0.374	0.317 – 0.431
	t_1		0.821 ms	0.373 - 1.269 ms
	b		0.733	0.688 -0.777
$\Phi_{Int\rightarrow TH}$		On-switching curve fit with 5-state photoswitching model	12.6 %	11.7 – 13.7 %
ε^{Int}_{405}		On-switching curve fit with 5-state photoswitching model	16555 M^{-1} cm^{-1}	15913 – 17226 M^{-1} cm^{-1}
α		On-switching curve fit with 5-state photoswitching model	0.642	0.631 – 0.653

The estimated parameters reported in Supplementary Table 3 were obtained from fitting the response of our 5-state photoswitching model to the experimental data. Nonetheless, given that such parameters are correlated and that there is a correction factor associated with the experimental 405 nm power density (estimated to be 0.642 ± 0.06), other combinations of $\Phi_{Int\rightarrow TH}$ and ε^{Int}_{405} would also reproduce the experimental curve with similar fidelity. We deemed that the best way to account for this source of experimental error is to study the correlation between the different parameters using the simulation tool. The resulting $\Phi_{Int\rightarrow TH}$ and ε^{Int}_{405} from the kinetic model fitting in Supplementary Figure 6 are to be understood in the context of a multiparametric family of solutions where certain experimental parameters that influence the kinetic model fit have an implicit uncertainty. As illustrated in Supplementary Note 2, a long-lived intermediate that can absorb 405 nm light will reduce the expected fluorescence signal at the second photoswitching cycle. The magnitude of this drop will be dictated by the 405 nm excitation dose, its spectroscopic properties (ε^{Int}_{405}, $\Phi_{Int\rightarrow TH}$) and the solution's pH. Given

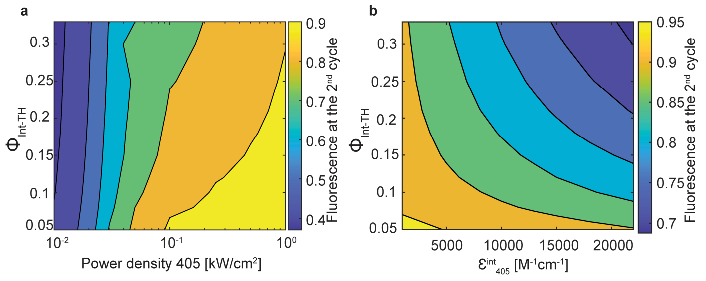

Supplementary Figure 7. (a) Normalized fluorescence at the 2nd cycle as a function of the $\Phi_{Int\rightarrow TH}$ and the 405 nm irradiation power density. (b) Normalized fluorescence at the 2nd cycle as a function of $\Phi_{Int\rightarrow TH}$ and ε^{Int}_{405}.

such multiparametric dependencies, there exist multiple combinations of parameters that can reproduce the same initial drop. Similarly to the kinetic model of Supplementary Figure 2b, all the surface plots included in Supplementary Figure 7 correspond to a 5-state kinetic scheme without triplet state nor active bleaching pathways as they do not have a significant influence when assessing the magnitude of the initial drop, which is defined as the normalized fluorescence signal at the second cycle in a fatigue recording. The off-switching time in all the plots of Supplementary Figure 7 was 1.2 ms and the 488 nm illumination power density was 0.2 kW/cm^2 and τ_{Int} = 825 µs.

In the case of Supplementary Figure 7a, we examined how changing the 405 nm excitation dose affected the initial drop for a series of $\Phi_{Int\rightarrow TH}$ while keeping $\varepsilon^{Int}{}_{405}$ = 16000 M^{-1}cm^{-1}. From the simulation result, we observe that at low 405 nm power densities (< 50 W/cm^2), the illumination dose will mostly dictate the loss of fluorescence between the 1st and 2nd cycles. As we increase the dose, such loss becomes smaller as a larger share of the ensemble population is on-switched. However, a C^{Int} with a more efficient $\Phi_{Int\rightarrow TH}$ (~ 0.30) is expected to have a loss of fluorescence at the second cycle of around 20%, while in a less efficient one, $\Phi_{Int\rightarrow TH}$ = 0.05 the loss would be around 10%. This closely relates to the discussion on the relaxation time in Supplementary Note 4, a higher $\Phi_{Int\rightarrow TH}$ will result in a bigger fraction of the ensemble being redirected back to the off-state where it would be trapped and not contribute to the photoswitching turnover, yielding a lower fluorescence signal. Supplementary Figure 7a also illustrates that for a given 405 nm power density the evolution of the initial drop with $\Phi_{Int\rightarrow TH}$ is rather smooth, with many different quantum yields resulting in similar losses of fluorescence at the second cycle.

The correlation between $\Phi_{Int\rightarrow TH}$ and $\varepsilon^{Int}{}_{405}$ is clearly visible in Supplementary Figure 7b. For a 405 nm illumination power density of 100 W/cm^2, a C^{Int} that very efficiently isomerizes back to the *TH* form (top right corner, high $\Phi_{Int\rightarrow TH}$ and $\varepsilon^{Int}{}_{405}$) have an expected normalised fluorescence at the second cycle around 0.7, while on the other end, one can on-switch around all the ensemble population if both $\Phi_{Int\rightarrow TH}$ and $\varepsilon^{Int}{}_{405}$ are rather low (left bottom corner). In between, a large family of solutions have the same expected initial drop as the two parameters appear to be anti-correlated.

Supplementary Note 4: Bleaching pathways in rsEGFP2

We systematically characterized the photoswitching fatigue of rsEGFP2 by investigating the influence of the illumination doses at 405 and 488 nm. We identified two main components for the loss of fluorescence upon thousands of on/off photoswitching cycles: a fast initial drop and a gradual loss of fluorescence across hundreds/thousands of cycles that will be called fatigue fraction (from the 4th to the 2000th cycle). Moreover, we observed that mainly the latter is modulated by both illumination wavelengths, thus, we focused on studying the fatigue fraction as a function of the power density of the two illuminations. Initially, we only consider the triplet state as the main bleaching pathway for rsEGFP2 – electronic states 1-12 in the kinetic scheme of Supplementary Figure 2b -, and within this model, we identify the photobleaching channels to stem both from the triplet and its first excited state. The addition of a photobleaching pathway was necessary to reproduce the 405 nm power density dependence observed in the experimental fatigue curves. Triplet state formation has traditionally been acknowledged as one of the main causes of photobleaching in organic dyes[20] as well as fluorescent proteins[8,11] since the triplet state can act as a reaction partner to molecular oxygen.

The experimental data points are shown in Supplementary Figure 8 below as dots (same as Figure 1d in the main text), the colour code represents the fatigue fraction (loss of fluorescence from the 4th to the 2000th cycle) for each combination of 405 and 488 nm power densities. Higher fatigue fraction values indicate a greater loss of fluorescence. Analogous to Figure 1d in the main text, the experimental data points are overlayed to the simulated values of the fatigue fraction across different illumination power densities for both wavelengths. In this case, the simulation only accounted for bleaching from the triplet and triplet excited states and from the colour coding it is readily observable that such a kinetic model cannot account for the increased loss of fluorescence of the experimental data at high 405 nm illumination doses. If the bleaching fraction derives only from the triplet state, the main driving force for photobleaching will be the 488 nm energy dose as this wavelength is preferentially absorbed by C^- ($\varepsilon^{on}{}_{488} > \varepsilon^{on}{}_{405}$) from which the triplet state will appear, moreover, the triplet state has also an absorption peak at 488 nm[7] that will contribute to an enhancement of photobleaching. In such a model, the 405 nm dose will mainly dictate the concentration of C^- and, as the available fraction of C^- saturates, so will the loss of fluorescence associated with that wavelength. Our experimental data differ from the behaviour of this model, i.e. an increase of the 405 nm illumination dose produces a continuous increase of the fatigue fraction.

To simulate the loss of fluorescence of rsEGFP2 upon thousands of photoswitching cycles for different combinations of 405 and 488 nm illuminations, we first identify the effective quantum yields of the different bleaching pathways active in the kinetic model for a ramp of 488 nm illumination doses at a given 405 nm power density (dark-shaded area in Supplementary Figure 8a). Using these data points,

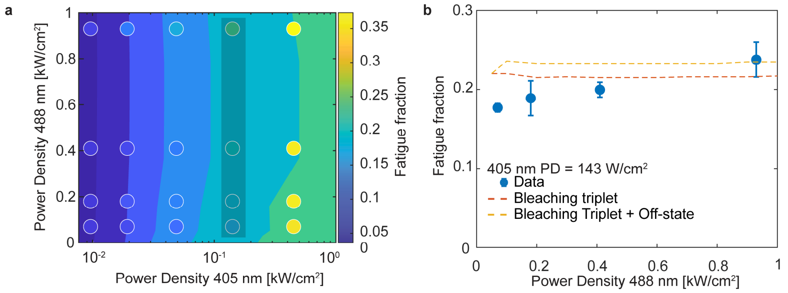

Supplementary Figure 8. (a) Fatigue fraction data and simulation with bleaching only from the triplet state. The data (coloured dots) predict a much higher fatigue fraction than the kinetic model at high 405 nm power densities. The data points in the dark-shaded rectangle (405 nm power density = 140 W/cm^2) were used to fix the effective bleaching quantum yields employed in the simulations. (b) Fatigue fraction data (blue) at 220 W/cm^2 compared to different bleaching models: only triplet state (orange) and triplet state plus off-to-on bleaching (yellow).

we find a combination of effective bleaching quantum yields that reproduce well the experimental fatigue curves, and then, using the same parameters, we extrapolate to the whole two-dimensional parameter space of illumination power densities. The same procedure was carried out to simulate the fatigue fraction presented in Figure 1d in the main text, in that case however, three effective bleaching quantum yields were considered as we included a bleaching pathway that appears in the off-to-on transition. To recreate the experimental conditions, the 488 nm off-switching time for a given power density was set such as the fluorescence signal had decayed to 20% of the initial value. Supplementary Figure 8b compares the experimental fatigue fraction (in blue) to the simulations given two different models, one considering triplet bleaching (orange dashed line) and the other considering triplet bleaching as well as off-to-on bleaching (yellow dashed line). The fatigue fraction including the off-to-on bleaching was slightly overestimated at low 488 nm power densities, however, the general trends of the fatigue fraction two-dimensional power density space were satisfactorily reproduced. The effective bleaching quantum yields used in the simulations are tabulated in Supplementary Table 4 below.

Supplementary Table 4. Effective bleaching quantum yields used in the fatigue fraction simulations.

**Bleaching model**	$\Phi_{Bleach\text{-}triplet}$	$\Phi_{Bleach\text{-}triplet\text{-}excited}$	$K_{Bleach\text{-}Off\text{-}to\text{-}On}$
**Triplet only**	1.3 x 10^{-3}	5 x 10^{-6}	-
**Triplet + Off-to-On**	0.9 x 10^{-3}	5 x 10^{-7}	4 ms^{-1}

As shown in Figure 1d in the main text, the addition of a bleaching pathway stemming from the off-to-on transition reproduced better the trends observed in the experimental data at high 405 nm power densities. We reason that the presence of the long-lived, high-absorbing C^{Int} results in light-induced quasi-equilibrium for TH, $CH^{\dagger}$ and C^{Int} during 405 nm illumination which yields inefficient on-switching and promotes photobleaching. Using the simulation tool, we can visualize the population evolution over time reflecting the exchange of population between TH and C^{Int} at the onset of the 405 nm pulse followed by the rise of the on-state, C^{-}. The accumulation of population of both TH and C^{Int} results in an increase of excitations per switching cycle for both species which becomes more significant for $C^{Int}*$ given that we describe the state as fluorescent with a nanosecond lived lifetime ($C^{Int}*$, $\tau = 1.6$ ns, $TH*$, $\tau = 20$ ps) as shown in Supplementary Figure 9b. Our data does not provide mechanistic insight on the nature of the photobleaching pathway and we rationalize the observed bleaching appearing in the off-to-on transition as lumped process that can be modelled with an effecting bleaching rate from TH, $CH^{\dagger}$ and C^{Int}, $K_{Bleach\text{-}Off\text{-}to\text{-}On}$, with a dominant contribution from the latter given its increased excited state lifetime.

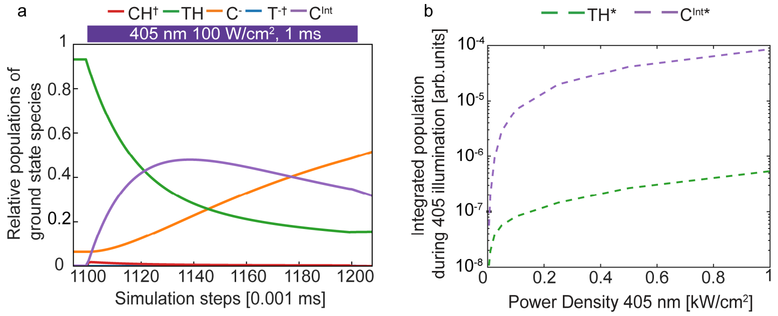

Supplementary Figure 9. (a) Relative populations of the relevant ground state species during the OFF-to-ON transition, $CH^{\dagger}$, TH, C^{-}, $T^{-\dagger}$ *and* C^{Int} in a 405 nm illumination period. (b) Integrated concentration of $TH*$ *and* $C^{Int}*$ per cycle during 405 nm illumination time as a function of the 405 nm power density.

We encountered variability in the observed initial drop in the photoswitching fatigue curves across different datasets. As shown in Supplementary Figure 7a the 405 nm power density will influence the magnitude of the initial drop, thus, divergences from the assumed experimental conditions may be behind the variability. Another experimental source of error might stem from the experiment procedure itself as the sample needs to be placed in focus for every measurement which implies a continuous illumination before the actual pulse scheme. Although this process is carried out at low irradiances (10 -100 µW), the variable illumination time may lead to differences in the expected response of the fluorophore. We tentatively investigated the influence of this initial perturbation on the expected initial drop for rsEGFP2 in the presence of different active bleaching channels: only from the triplet state or both the triplet and the off-to-on photoswitching. We compared the effect of initial perturbation to the expected response of a simulation without previous illumination. Note that the latter condition was the standard for all the simulations presented in the text. The perturbation was simulated by adding a pulse of both 405 and 488 nm illumination with variable length and power densities before the photoswitching fatigue curve simulation.

In Supplementary Figure 10a, we simulated the expected fluorescence at the second photoswitching cycle with different bleaching pathways active in the modelling of rsEGFP2. The green dashed line in Supplementary Figure 10b represents the expected initial drop if no perturbation is applied and no bleaching channels are assumed. The input pulse scheme in the simulation was a 1 ms dose of 405 nm light at 100 W/cm^2 and 1.5 ms of 488 nm at 150 W/cm^2. The green dashed line represents the maximum signal available at such illumination doses. Even if no photobleaching channels are active, a small perturbation of 50 mJ/cm^2 reduces the fluorescence output at the second cycle by around ~ 3% (no bleaching channels, blue curve). This reduction is enhanced further if the initial perturbation dose increases. The addition of the bleaching channels into the modelling (triplet bleaching, orange curve and triplet + off-to-on bleaching, yellow curve) implies a larger fluorescence drop in the 2nd cycle, reaching an ~ 8% fluorescence loss if all channels are active and the perturbation dose is 50 J/cm^2 (yellow curve) which is similar to the disagreement we observe between simulation and experimental data in Supplementary Figure 10b.

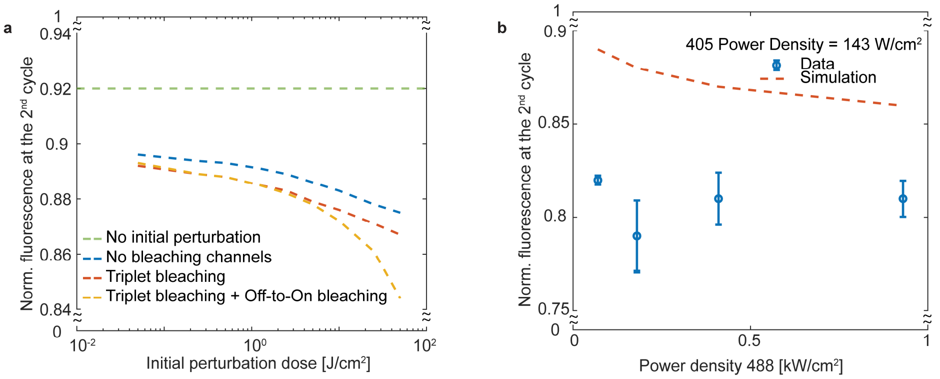

Supplementary Figure 10. (a) Effect of an initial perturbation on the initial drop with different models. The simulations were carried out at 405 nm 100 W/cm^2 and 488 nm power density 150 W/cm^2 with 1.5 illumination time. If all the bleaching channels are active, the initial drop decreases by around 8 % for a 50 J/cm^2 energy dose. (b) Experimental data and simulated initial drop as a function of the 488 nm power density for a 405 nm power density of 143 W/cm^2. There is a disagreement of around 6 % between data and simulation.

Supplementary Note 5: Thermal relaxation of long-lived dark states in rsEGFP2

To rationalize the data in Figure 1g in the main text, we have incorporated into our kinetic model an additional reversible dark state that forms from the triplet state. The dark state can thermally relax back to the emissive state (*Cis*$^-$ in Supplementary Figure 10a) or evolve further into a bleached state. We have kept all other photophysical pathways depicted in Supplementary Figure 2b. The insertion of this additional long-lived dark state was necessary to reproduce the data in Figure 1g in the main text since the observed thermally driven fatigue recovery occurred in a timescale much longer than the triplet state lifetime (~ 5 ms). Aided by the simulation tool, we reproduced the experiment of Figure 1g and performed a least-square fitting routine to determine the dark state lifetime (τ_D), $\Phi_{T1\text{-}to\text{-}dark}$ and $\Phi_{Bleach\text{-}dark}$ that best matched the data, by fixing all the other parameters from the previous experimental data. We found that the relaxation time of the dark state in the proposed kinetic model should be ~ 17 seconds (Supplementary Figure 11b and Supplementary Table 5). The value is in agreement with other accounts of reversible photobleaching evolving the second timescale[23], both in organic dyes[24] and fluorescent proteins[25,26]. Although the long lifetime hints to a radical state[21,22], our data cannot provide much insight into the spectroscopic properties of the long-lived dark state.

The phenomena observed in Figure 1g in the main text and Supplementary Figure 11b suggest that the dark state will be populated in a significant fraction, nonetheless, we set out to investigate how this reversible photobleaching will be reflected in our measurements given that the relaxation time of the dark state is almost as long as the experiment total time (e.g. with a total dwell time of 10 ms and 1680 on/off cycles the experiment time is also ~ 17 seconds).

From the kinetic model, the concentration of the dark state shows a continuous build-up (Supplementary Figure 12). With a longer waiting time the dark state has more time to relax, and a lower steady-state level is reached. Under the main characterization condition of the presented work, the dark lifetime (~17 s) is in the same order of magnitude as the experimental time (~ 20 s). The dark state bleaching and recovery will progress slowly during the experiments, on average such dark states species will be a passive player during the experiment mimicking a bleaching species. Accordingly, in the minimal kinetic model presented here, the additional dark state pathway is effectively lumped with irreversible photobleaching. Overall, this will result in a reasonable representation of the overall kinetic behaviour with some small biases on the specific kinetic constant related to the triplet branch. It is important to

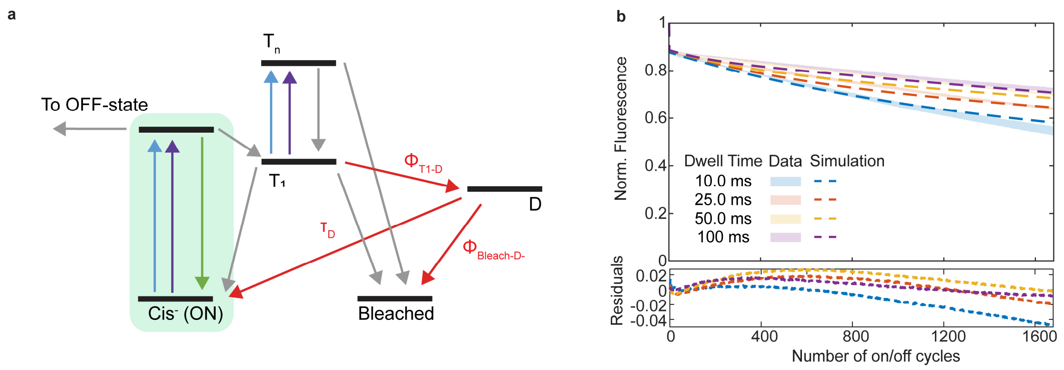

Supplementary Figure 11. (a) Schematic representation of the photophysical scheme accounting for the formation of long-lived dark states (*D*) via the first triplet excited state (*T1*). Similarly to the kinetic model proposed for IrisFP[21,22], the long-lived dark state can relax back to the emissive state (*Cis*$^-$) or further evolve to a photobleached state. The red coloured arrows indicate the newly added photophysical pathways and the fitted parameters (τ_D, $\Phi_{T1\text{-}to\text{-}dark}$ and $\Phi_{Bleach\text{-}dark}$). (b) Experimental data and simulation of photoswitching fatigue thermal recovery. For this experiment, the 405 nm dose was 1 ms and 240 W/cm^2 while the 488 nm dose was 5 ms and 40 W/cm^2. The dotted lines represent the simulation while the shaded areas are ± σ of the experimental data. Below, are the residuals of each experimental curve as *data-simulation*.

note that co-illumination with light above 592 nm completely bypasses the dark state formation by directly accessing the triplet state, the precursor of any longer-lived dark state. Because of the strong correlation of the triplet and radical parameters, attempting to fit the expanded kinetic model simultaneously will increase the uncertainty of the parameters, weakening the presented effective kinetic model.

Considering the radical lifetime could provide practical guidelines in imaging experiments. In widefield microscopy, slower acquisition speeds (< 0.05 Hz) will minimize the build-up of substantial steady-state radical population, whereas faster speeds (> 0.05 Hz) will maximize it. In confocal imaging, the scanning inherently introduces dark intervals of 20–100 ms or more, with parallelized confocal and RESOLFT systems providing shorter dark times than point-scanning approaches. However, the radical lifetime in biological samples could be shorter than our experiments in PAA and difficult to predict a priori. Moreover, in live-cell experiments, acquisition timing is dictated by the dynamics of the biological process rather than by RSFP photophysics.

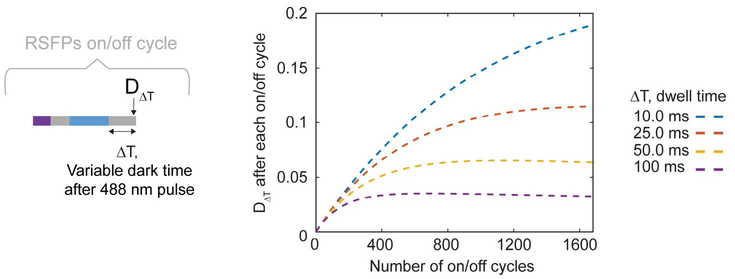

Supplementary Figure 12. (a) *D* population at the end of each photoswitching cycle pulse scheme. For a total dwell time of 10 ms (blue curve), the population of long-lived dark species continuously increases, while for longer total dwell (25 ms – orange, 50 ms – yellow and 100 ms – magenta) times the long-lived dark state population reaches a saturation level.

Supplementary Table 5. Parameters used in the simulations of Supplementary Figures 11, 12.

Simulation	405 nm dose	488 nm dose	Radical lifetime	$K_{Bleach\text{-}OFF}$	$\Phi_{Bleach\text{-}triplet}$	$\Phi_{Bleach\text{-}triplet\text{-}excited}$	$\Phi_{T1\text{-}to\text{-}radical}$	$\Phi_{Bleach\text{-}radical}$
**Thermal recovery**	1.0 ms	5.0 ms	16641	2 ms^{-1}	0.6 x 10^{-3}	1.0 x 10^{-7}	0.18016 %	1.76x10^{-3}
	240 W/cm^2	40 W/cm^2	c.i. (95%) 15936 - 17377				c.i. (95%) 0.18011 – 0.18021	c.i. (95%) 3.01x10^{-6} – 1.03

Supplementary Note 6: On-switching dynamics in rsEGFP2

Complete photoswitching, i.e. moving 100% of the fluorophore's population from the on to the off state and vice versa, is in general not possible since both on and off states present non-zero absorption cross-sections for each of the wavelengths used in photoswitching[19]. In other words, the wavelength used to trigger the *off-to-on* transition can also be absorbed by the on-state and promote the *on-to-off* transition equilibrating the off-state population to a small fraction after on-switching. The presence of an intermediate in the photocycle, like in rsEGFP2, adds a temporal dimension to this effect since the interplay between the excitation rate and the lifetime of the intermediate state will dictate how big the fraction that equilibrates is.

As introduced in Supplementary Notes 2 and 3, the presence of the long-lived intermediate state is responsible for the initial drop observed in the photoswitching fatigue experiments. This sharp decrease in the expected fluorescence of rsEGFP2 after photoactivation is a result of the light-induced quasi-equilibrium that is established between *TH*, $CH^{\dagger}$ and C^{Int} if the 405 nm illumination time is comparable to the lifetime of the intermediate state while illuminating the sample with typical 405 nm illumination intensities (~ 50 – 500 W cm^{-2}).

The nonlinearity of the on-switching transition also means that delivering the same total illumination dose in different manners – longer and less intense pulses rather than high-intensity bursts – yields a different on-switching efficiency. As shown in Supplementary Figure 13a below, the relative population of *TH* just before the 488 nm pulse starts decreases if the previous on-switching transition is triggered by longer pulses, while the inverse behaviour is observed in the simulation for C^{-}. While delivering the 405 nm on-switching photons slower (longer 405 nm illumination times) the probability of C^{Int} transitioning back to *TH* is reduced and the turnover between the off and the on state (*TH* and C^{-} respectively) is maximized which will result in more fluorescence photons collected during the 488 nm read-out pulse. It is important to note that the C^{Int} concentration before 488 nm illumination in Supplementary Figure 13a is constant and close to 0 because the simulated pulse scheme includes a long resting time (> 3 ms) between 405 and 488 nm pulses which should allow the relaxation of any C^{Int} population into the fluorescent state, C^{-}. On the other hand, if the on-switching dose is delivered very rapidly (< 100 μs illumination time and > 1 kW cm^{-2}) the concentration of $CH^{\dagger}$ after photoactivation increases. An accumulation of $CH^{\dagger}$ will lead to an increment in *TH* concentration as the isomerization reaction between these two states is very probable, shifting the equilibrium away from C^{Int} and, therefore, reducing the available concentration in C^{-}. When the illumination dose is delivered

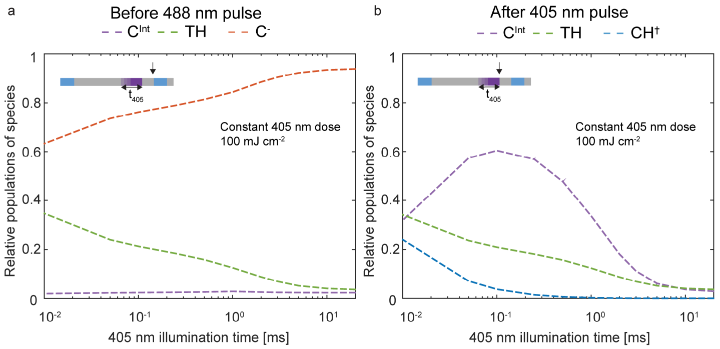

Supplementary Figure 13. (a) Relative populations of states C^{Int}, *TH* and *C-* just before the 488 nm illumination. The simulated pulse is shown in the inset. The 405 nm illumination time was variable – from 0.01 to 20 ms – while the energy dose was kept constant at 100 mJ cm^{-2} – the corresponding power densities ranged from 10^4 to 5 W cm^{-2} –. The 488 nm dose was 200 W cm^{-2} and 1.2 ms illumination times in all the simulated points. (b) Relative populations of states C^{Int}, *TH* and $CH^{\dagger}$ just after the 405 nm illumination. The simulated pulse is shown in the inset. The 405 nm illumination time was variable – from 0.01 to 20 ms – while the energy dose was kept constant at 100 mJ cm^{-2} – the corresponding power densities ranged from 10^4 to 5 W cm^{-2} –. The 488 nm dose was 200 W cm^{-2} and 1.2 ms illumination times in all the simulated points.

very slowly the photoswitcher will act closer and closer to a simple on/off switch without visible effects and accumulation of intermediate states, i.e. on the right side of Supplementary Figure 13a-b the population resides almost fully in the on-state, C^{-}.

Complementarily, we verified in live cells if the modulation in time of the 405 nm illumination dose can be advantageous in a microscopy setting. Briefly, we applied two sequential pulse schemes to the same U2OS cells, labelled with rsEGFP2-ActinChromobody, in our parallelized confocal microscope. Firstly, a low 405 nm power density (14 W/cm^2) was used for photoactivation for 20 ms followed by a 1 ms 488 nm pulse (0.5 kW/cm^2) to acquire the fluorescence signal, we deemed this pulse scheme as *slow image* (Supplementary Figure 14a). Once the confocal frame was acquired, the same cell was imaged with another pulse scheme we named *fast image* (Supplementary Figure 14b) consisting of high intensity (1.05 kW/cm^2) but short 405 nm illumination time (0.25 ms) after which a 488 nm pulse (0.5 kW/cm^2 and 1 ms) was used as the read-out step. To avoid photobleaching bias from one of the on-switching doses, we alternated which of the pulse schemes (*slow or fast images*) was first seen by the cell, i.e. *cell 1* was imaged with the *slow image* pulse scheme first, *cell 2* was first imaged the *fast image* pulse scheme instead.

Under these illumination conditions, the total energy dose delivered by the 405 nm is roughly the same (~ 280 mJ/cm^2), however, extending the illumination time at a low 405 nm power density can enhance the fluorescence collected in the read-out step as shown in Supplementary Figure 14. We quantified the effect by computing the intensity ratio per pixel (*Slow Image / Fast Image*) for each cell. As seen in Supplementary Figure 14c,d, most of the segmented pixels in the image show a pixel value over 1, indicating that a higher fluorescence signal was captured when imaging with the *Slow Image* pulse scheme. This behaviour is consistent with our observations of rsEGFP2 in the PAA layer. Our results in

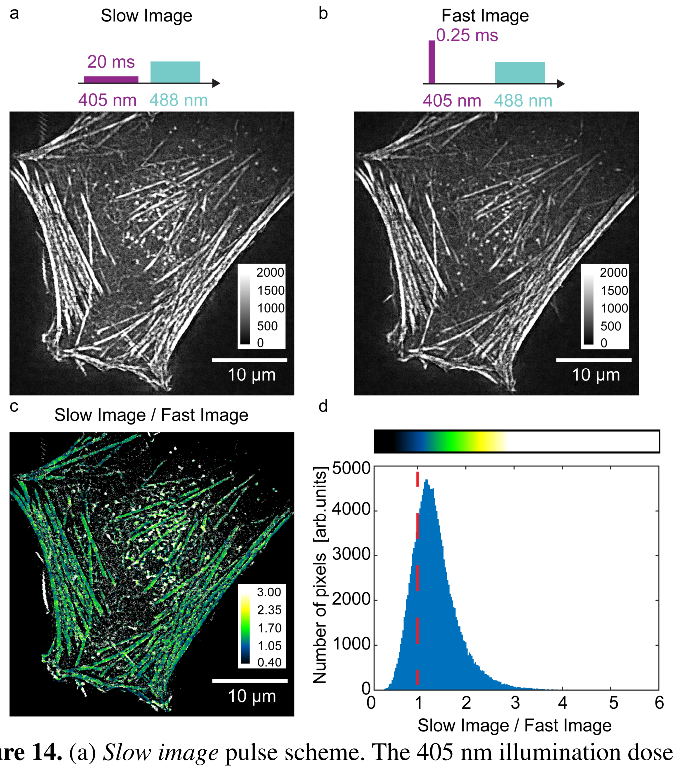

Supplementary Figure 14. (a) *Slow image* pulse scheme. The 405 nm illumination dose was set to 20 ms and 14 W/cm^2, and the fluorescence was captured with a 488 nm read-out pulse (500 W/cm^2 and 1 ms). (b) *Fast image* pulse scheme. The 405 nm illumination dose was set to 0.25 ms and 1.05 kW/cm^2, and the fluorescence was captured with a 488 nm read-out pulse (500 W/cm^2 and 1 ms). (c) Ratiometric image (*Slow image / Fast image*). The pseudocolour of each pixel in the actin fibres shows the value of the intensity ratio. (d) Histogram of the pixel values in (c). The colour bar on top represents the pseudocolour associated with each bin in the histogram.

live-cell show that modulating the 405 nm dose in time is a viable route for maximizing the photon budget of rsEGFP2 in an imaging setting, nonetheless, this comes at the compromise of lower temporal resolution.

Supplementary Note 7: Parametrization of the triplet state of rsEGFP2

To fully understand the photoswitching fatigue recovery we observed in rsEGFP2, it became apparent that we needed to include some of the triplet's spectroscopic properties. Firstly, we investigated the triplet's decaying time with µs temporal resolution in our custom-built confocal microscope using a three-pulse illumination sequence, 405-488-488 nm. In these experiments, a first burst of 405 nm light (5 µs and 30 kW/cm^2) prepares the protein's ensemble population in the on-state, after a delay of 3 ms, a 488 nm dose (500 µs and 40 kW/cm^2) will off-switch the protein's population within the confocal volume. After a variable delay (from 5 µs to 500 ms) a second 488 nm dose, identical to the first one, will interrogate the same volume. The fluorescence emitted by the sample is recorded and the area below the second off-switching curve is monitored and compared to the expected fluorescence for a 0 ms delay control experiment, we call this parameter the ratiometric signal, i.e. the ratio between the integrated fluorescence signal from the second 488 nm pulse with and without a dark waiting time. As shown in Supplementary Figure 15, we observe an increase in the fluorescence signal of the second off-switching pulse as a function of the delay between the 488 nm pulses, moreover, we reason that such an increase comes from the relaxation of a dark state generated during the first 488 nm illumination pulse. Additionally, we fitted the dependence of the ratiometric signal to the delay between 488 pulses to a monoexponential fit yielding a raising time τ_1 = 2.46 ms (Figure 2c in the main text) which is consistent with the reported triplet lifetime of rsEGFP2[7].

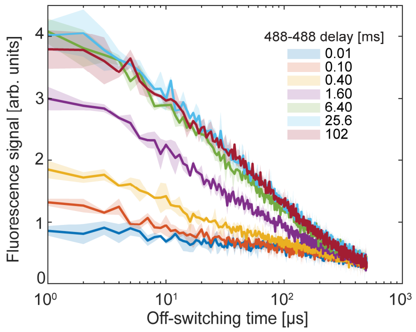

Supplementary Figure 15. (a) Off-switching curve during the second 488 nm pulse for multiple delay times. The fluorescence signal increases with the delay until it saturates for delays longer than 6.4 ms. The curves are built from the mean decay ± σ (shaded area) from at least 3 regions in the PAA gel.

Supplementary Table 6. Parameters of biexponential fit of Figure 2b in the main text.

Parameter		Method	Estimated value	Confidence interval (95%)
y = a – b*exp(-t/t$_{T1}$)	***a***	Monoexponential fit	2.046	2.020 – 2.071
	b		0.978	0.943 – 1.013
	t$_{T1}$		2.461 ms	2.134 – 2.787 ms

We characterised Φ_{RISC} from the triplet excited state to the singlet state from our photoswitching fatigue recovery data using a 592 nm illumination power density ramp at 488 nm illumination dose 1.5 ms and 200 W/cm^2. Additionally, we fixed the effective bleaching quantum yields from all the active channels to reproduce the data without 592 nm illumination: $\Phi_{Bleach\text{-}triplet}$, $\Phi_{Bleach\text{-}triplet\text{-}excited}$, $\Phi_{Bleach\text{-}TH}$ which resulted in Φ_{RISC} = **0.25 %**. Comparatively, we used such reverse intersystem crossing quantum yield in another 592 nm power density ramp with a higher 488 nm illumination power density (420 W/cm^2 and 0.9 ms illumination time) and tried to reproduce the photoswitching fatigue curves with its corresponding set of effective bleaching quantum yields. The results of the simulations are shown in Supplementary Figure 16, where the dotted lines represent the simulated photoswitching fatigue curves while the shaded areas are ± σ of the experimental data. Despite the experimental and modelling challenges in reproducing photobleaching data, we believe there is a good agreement between kinetic

model predictions and experiments. Overall, the output of the kinetic model can grasp the coarse behaviours of the data. For both experiments, the simulation tends to display a lower photoswitching fatigue recovery at lower 592 nm power densities, especially at 610 W/cm^2, possibly due to an underestimation of the 592 nm illumination power, nonetheless, once the effective bleaching quantum yields are fixed the simulation is consistent in reproducing the control curve and the trends suggested by the data.

It is important to note that experimental bleaching depends upon several factors regarding sample preparation, e.g. the diffusion properties and amount of oxygen dissolved in the sample. For this reason, we optimized the bleaching quantum yield for each dataset in order to reproduce the observed trends in the data. The quantum yield for RISC from a given higher triplet state in a specific solvent should be conserved as it is a molecular parameter, and we argue that it should be conserved in the modelling of all the experimental data.

The Φ_{RISC} value that best reproduced the trends observed in the 592 nm power density ramp is around 2-fold higher than the one reported in a study published on rsEGFP2 at 100K[7]. As mentioned, the power was measured at the objective's back aperture to calculate the power density and the laser power delivery over time was assumed constant, nonetheless, given the variability of the experimental data we estimate an error within 20 % of the measured power density. In that regard, one could explain our

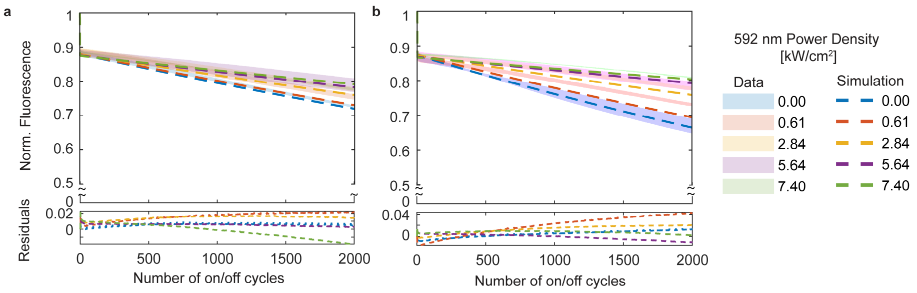

Supplementary Figure 16. (a) Experimental data and simulation of a power ramp of 592 nm at 488 nm = 1.5 ms and 200 W/cm^2. The dotted lines represent the simulation while the shaded areas are ± σ of the experimental data. Below, are the residuals of each experimental curve as *data-simulation*. (b) Experimental data and simulation of a power ramp of 592 nm at 488 nm = 0.9 ms and 420 W/cm^2. The dotted lines represent the simulation while the shaded areas are ± σ of the experimental data. Below, are the residuals of each experimental curve as *data-simulation*.

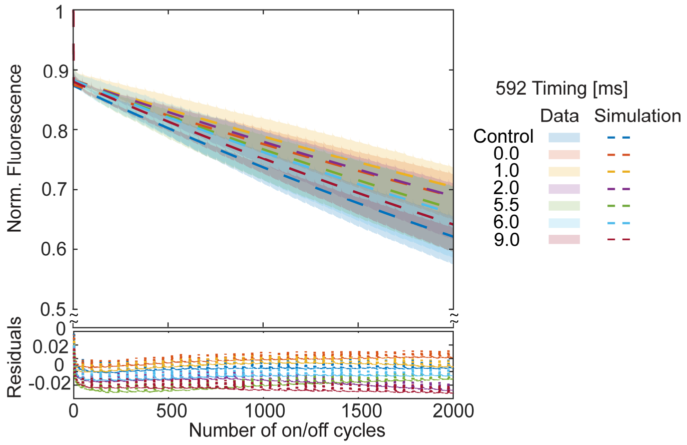

Supplementary Figure 17. Experimental data and simulation of a power ramp of photoswitching fatigue curves with the addition of 592 nm illumination. The 592 nm pulse was incorporated into the pulse scheme with an added delay to the 488 nm dose. The dotted lines represent the simulation while the shaded areas are ± σ of the experimental data. Below, are the residuals of each experimental curve as *data-simulation.*

overestimation of the Φ_{RISC} magnitude due to an underestimation of the 592 nm power density at the sample plane.

A similar procedure was carried out when reproducing the photoswitching fatigue data as a function of the delay between the 488 and 592 nm pulses. The parameter was set to Φ_{RISC} = **0.25 %** and a set of effective bleaching quantum yields was found to properly reproduce the data. Similarly, Supplementary Figure 17 shows a comparison of the simulations (dotted curves) and ± σ of the experimental data (shaded areas). The colour coding represents the different conditions. All the relevant parameters used in the simulations of Supplementary Figures 16 and 17 are shown in Supplementary Table 7.

Supplementary Table 7. Parameters used in the simulations of Supplementary Figures 16 and 17. Note that the illumination doses are the same as in the experiments.

Simulation	405 nm dose	488 nm dose	592 nm dose	$K_{Bleach-OFF}$	$\Phi_{Bleach-triplet}$	$\Phi_{Bleach-triplet-excited}$	Φ_{RISC}
**592 nm power ramp, low 488 power**	1.0 ms<br>230 W/cm^2	1.5 ms<br>200 W/cm^2	1.0 ms<br>0 – 7.4 kW/cm^2	0.6 ms^{-1}	0.8 x 10^{-3}	1.0 x 10^{-7}	0.25 %
**592 nm power ramp, high 488 power**	1.0 ms<br>200 W/cm^2	0.9 ms<br>420 W/cm^2	1.0 ms<br>0 – 7.4 kW/cm^2	0.7 ms^{-1}	1.1 x 10^{-3}	1.0 x 10^{-7}	0.25 %
**488 – 592 nm delays**	1.0 ms<br>170 W/cm^2	1.0 ms<br>250 W/cm^2	1.0 ms<br>7.0 kW/cm^2	5 ms^{-1}	1.0 x 10^{-3}	4.0 x 10^{-7}	0.25 %

Supplementary Note 8: Higher excited triplet states and RISC mechanism

The proposed photophysical mechanism for rsEGFP2 described in Supplementary Note 1 includes a pathway to account for the observed light-induced photoswitching fatigue recovery in the data. We rationalized the effect as an optical depopulation of the C^- triplet state ($^3C^-$ in Supplementary Figure 2b). In our model, the absorption of 592 nm leads to an excited triplet state from which it is possible to access the excited state of the singlet form (C^-*) via RISC. In recent years, the properties of the triplet state in fluorescent proteins have been studied spectroscopically, allowing us to have a certain clarity on where such states are in the energy ladder[8] or which are their absorption properties[7,8]. However, they lack conclusive evidence for which is the precise mechanism that leads to the repopulation of the singlet form via RISC. In the literature, the effects of light-induced RISC in EGFP[11] and rsEGFP2[12] mainly assume that any conversion of population from the triplet to the singlet will occur via the excited states. In describing rsEGFP2, we make a similar assumption. The existence of such a pathway in fluorescent proteins has been proven and observed by means of optically-activated delayed fluorescence (OADF)[13,14] where a fluorescence photon is measured after illuminating the sample with longer wavelengths respect to the fluorescence excitation dose and that is delayed in time. This phenomenon is only possible if the RISC mechanism involves the excited states from the singlet manifold, and although OADF has never been observed in rsEGFP2, it cannot be a priori excluded as a plausible mechanism to explain the photoswitching fatigue recovery in our data.

Nonetheless, we acknowledge that other photophysical pathways might be in place to account for the observed light-induced recovery, for example, RISC can be induced from excited triplet states into the vibronic manifold of S0 as the density of vibronic states increases exponentially with the excitation energy. Using the simulation tool, we explored whether channeling RISC $Tn \rightarrow S_1$ or $Tn \rightarrow S_0$ would have an impact on the light-induced recovery. As shown in Supplementary Figure 18a, absorption from different wavelengths of light will populate different states within the triplet excited state energy ladder, from there the light-induced repopulation of the emissive form can occur via S_1 (Supplementary Figure 18b) or S_0 (Supplementary Figure 18c) due to RISC followed by intramolecular vibrational relaxation (IVR), we assume the IVR step is instantaneous. In our simulations, we see no sensible impact of the RISC pathway on the expected light-induced recovery. In essence, our kinetic model is built from

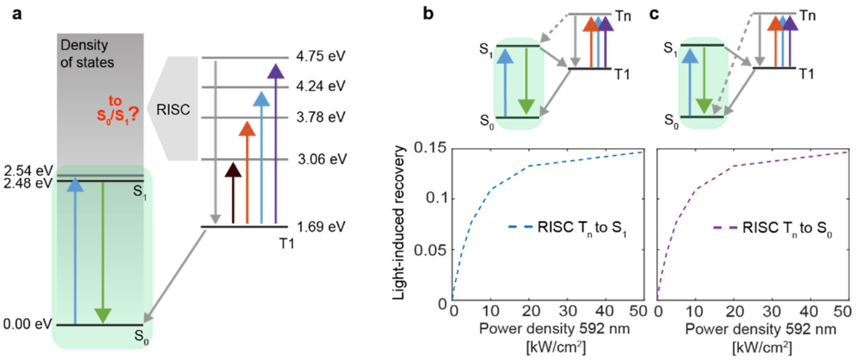

Supplementary Figure 18. (a) Schematic of energy ladder of singlet and triplet states for rsEGFP2 in eV. The position of S_1 was determined from the maximum in the emission spectra[9]. The position of T_1 was determined from the maximum in phosphorescence emission spectra of EGFP[8] as an approximation of the energy level of the first triplet state of rsEGFP2. The energy levels of the subsequent excited triplet states were calculated as $E(\lambda) = 1.69 + 1.24/\lambda$, with λ in μm. The coloured arrows indicate which wavelength would populate each of the energy levels: 3.06 eV, 900nm; 3.78 eV, 592 nm; 4.24 eV, 488 nm; 4.75 eV, 405 nm. Line arrows indicate the different RISC mechanisms that were simulated. (b) Light-induced photoswitching fatigue recovery as a function of the 488 nm power density and 592 nm power density. The RISC mechanism of the simulation is the transfer of population from T_n-toS_1. (c) Light-induced photoswitching fatigue recovery as a function of the 488 nm power density and 592 nm power density. The RISC mechanism of the simulation is the transfer of population from T_n-to-S_0. All the simulations were carried out at 1 ms 405 nm and 100 W/cm^2 power density.

effective parameters to describe the effects observed in the experimental data within a reasonable photophysical framework.

On a similar note, we simplified the character of all excited triplet states that can be accessed by light into a single effective state T_n with the absorption probability weighted by the reported ε_λ from rsEGFP2's triplet state absorption spectra[7]. Within our effective kinetic model description, we can represent T_n and its effect on photobleaching and photoswitching fatigue recovery with just a few parameters: Φ_{RISC}, RISC quantum yield; Φ_{Bleach}, bleaching quantum yield; τ, excited state relaxation time. We acknowledge this is an oversimplification in the characterization of the system and, in particular, when describing excited triplet states that are accessed with different wavelengths it is plausible that each of these states respond very differently in terms of Φ_{RISC} and Φ_{Bleach}. However, we argue that distilling all these different behaviours into an effective parameter provides a reasonably good explanation for the observed trends in the experimental data while keeping the number of modelled states to a minimum.

Supplementary Note 9: Photoswitching fatigue recovery upon 592 nm illumination

Fatigue recovery simulations

The photoswitching fatigue recovery observed upon 592 nm illumination occurs because the 592 nm dose can be efficiently absorbed by the triplet state, but not by the singlet state, in addition to the non-zero probability of undergoing reverse intersystem crossing. Accordingly, the magnitude of the photoswitching fatigue recovery induced by the 592 nm co-illumination will depend on two factors: i) how big the photoswitching fatigue for a set of 405 and 488 illumination conditions is and ii) how much of the bleaching fraction can be recovered by the 592 nm dose.

For such purposes we used the simulation tool and studied the expected photoswitching fatigue recovery as a function of the illumination doses from the three wavelengths: 405, 488 and 592 nm. In the simulations, the 405 nm illumination time was kept constant at 1 ms, while the power density was fixed at 100 W/cm^2 when studying the dependencies of the recovery to 488 and 592 nm power densities. On the other hand, the 488 nm illumination time was tuned to off-switch the fluorescence by 80% when studying the dependencies of the fluorescence excitation wavelength and 592 nm, and the 488 nm illumination dose was kept at 1.2 ms and 200 W/cm^2 when investigating the effect of the 405 nm illumination. The bleaching parameters for the three active channels were identical to the 488-592 nm delay time simulation from Supplementary Figure 19.

The expected photoswitching fatigue recovery is relatively low at small 405 nm doses since there is no apparent bleaching, moreover, it is also noticeable that a threshold of ~ 2 kW/cm^2 of 592 nm power density should be surpassed to have approximately 5% of fatigue recovery. The 592 nm co-illumination recovery is favoured at higher 405 nm power densities (> 200 W/cm^2) when the C^- concentration is maximum, and high 592 nm energy doses as well, since more absorption per illumination dose will occur. The magnitude of the recovery, however, evolves slower as we move towards the upper right corner of the plot in Figure 2h in the main text (high 405 and 592 nm power densities) as the off-to-on bleaching becomes more dominant and the triplet concentration saturates.

Similarly, we also investigated the magnitude of the fatigue recovery as a function of the 488 and 592 nm power densities as displayed in Figure 2i in the main text. The photoswitching fatigue recovery is below 5 % until 592 nm power density ~ 1 kW/cm^2 for low 488 nm doses. The recovery is enhanced at a high 488 nm power density, although it reaches a plateau > 600 W/cm^2 for 592 nm power densities > 10 kW/cm^2 which may indicate the excitation saturation of the triplet state with 592 nm co-illumination. As the energy dose (illumination time × illumination power density) is roughly constant throughout the range of power densities investigated, the adverse effects from the triplet's high absorption at 488 nm are diminished and the magnitude of the recovery is mainly driven by the triplet state 592 nm excitation probability.

Comparison of fatigue recovery fractions and experiment with waiting times

The importance of the light-induced photoswitching fatigue recovery is readily observable if one attains the results shown in Figure 1g in the main text where the fatigue diminishes as the total pulse length increases. In that experiment, the illumination doses for all the different studied pulses were the same and only the dark time (no illumination of the sample) was successively increased. By increasing the pulse length of an order magnitude, the photoswitching fatigue was reduced by ~ 18 % after 1600 on/off cycles, however, if such a pulse scheme were implemented in an imaging context the temporal resolution would be heavily compromised.

Supplementary Figure 19a compares two approaches for fatigue recovery after 1600 cycles: increasing pulse length and co-illumination with 592 nm light. The horizontal lines in Supplementary Figure 19a represent the normalized fluorescence intensity recovery by increasing the waiting time per pulse compared to the standard 10 ms pulse, while the orange dots show the recovery experience by co-

illuminating with 592 nm in a standard 10 ms dwell time. Notably, co-illumination with 592 nm achieves similar fatigue recovery to longer pulses but preserves temporal resolution. These results demonstrate that while longer pulses effectively reduce photoswitching fatigue, co-illumination provides a more practical solution for imaging by minimizing bleaching effects without sacrificing time resolution.

Parameter dependency allowing fatigue recovery in the kinetic model

The kinetic model of rsEGFP2 developed in this work is based on several simplifications regarding the specific cyclic nature of the fatigue experiments and the specific parameters of the protein. Predictions based on the kinetic model need to be taken within the working assumptions. Briefly the main simplifications are: (i) the excited triplet states are modeled with wavelength independent bleaching and RISC quantum yields; (ii) the pH equilibriums are not considered because the equilibriums are very unbalanced towards C^- and *TH*; (iii) only the main source of triplet state (C^-) is considered. Simplifications are mainly driven by the lack of parameters available in literature and/or because our data would not be able to sustain more free parameters when fitting the fatigue fractions, without adding large uncertainty to correlated parameters.

Within the simplification of our kinetic model of rsEGFP2, fatigue recovery with 592 nm light co-illumination is observed when bleaching from the excited triplet state is not the dominant contribution and RISC is more probable. Here we discuss and explore the behaviors of the kinetic model as a function of the ratio of the bleaching quantum yields from T_1 and T_n.

The triplet state has been identified as the source of photobleaching in EGFP[8], moreover, recent reports highlight the importance of accounting for the excited triplet state given that rsEGFP2's triplet state presents a broad absorption peak at 488 nm[7]. In the absence of 592 nm co-illumination (Figure Supplementary 18b, blue line), if both bleaching from T_1 and from T_n have similar quantum yields, the fatigue fraction increases (left side of the plot) compared to when bleaching from T_1 is dominant (right side of the plot). With 592 nm co-illumination (orange line), we can observe the possibility of recovery only when bleaching from T_n is small enough and does not outcompete RISC. In our specific parametrization of rsEGFP2 this is valid when Φ_T / Φ_{Tn} is greater than approximately 600. Thus, reasoning within the assumptions of the kinetic model, our data suggest that in rsEGFP2 photobleaching

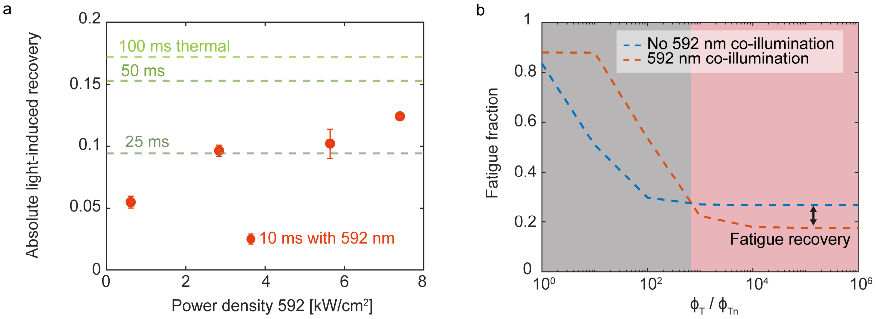

Supplementary Figure 19. Comparison of light-induced fatigue recovery to thermal fatigue recovery from Figure 1g. The horizontal lines correspond to the difference of the normalized intensity after 1600 cycles for a given pulse length to the standard 10 ms pulse. Longer pulse lengths yielded greater recovery. In orange, is the experimental data of fatigue recovery after 1600 cycles for a 488 nm power density of 420 W/cm^2. (b) Fatigue fraction with or without 592 nm co-illumination as a function of the ratio of bleaching quantum yields from the triplet ground state (Φ_T) and the triplet excited state (Φ_{Tn}). If the ratio $\Phi_T / \Phi_{Tn} < 600$ there is an increase in the simulated fatigue fraction, moreover, the addition of 592 nm co-illumination will enhance the photobleaching. If, on the other hand, bleaching mainly occurs from the triplet ground state ($\Phi_T / \Phi_{Tn} > 600$), adding the 592 nm light can decrease the photoswitching fatigue and promote the light-induced recovery we observe in our data. The simulations were carried out by fixing $\Phi_T = 10^{-3}$ and varying Φ_{Tn} accordingly. The 592 nm co-illumination dose was 4 kW/cm^2.

from the excited triplet state is not the dominant contribution allowing fatigue recovery when 592 nm light is applied.

Supplementary Note 10: Photoswitching fatigue recovery upon NIR illumination

The light-induced recovery was measured with co-illumination in the near-infrared (NIR) spectral region by taking advantage of the Ti: Sapphire tunable laser and the confocal microscope depicted in Supplementary Figure 29. Based on a recently published work, we tuned the longer wavelength co-illumination to 900 nm where the most prominent absorption peak of the triplet state of rsEGFP2 should be[7], and 810 nm, where the absorption of the triplet state for rsEGFP2 is similar to that at 592 nm. The experiments were carried out in a confocal microscope in a PAA-embedded rsEGFP2 sample and the protein was switched on and off by consecutive 405 and 488 nm pulses. As shown in Supplementary Figure 20a, the normalized light-induced recovery of the fluorescence signal after ~ 2000 photoswitching cycles was evaluated as a function of the longer wavelength co-illumination power density. We observed that for power densities > 40 kW/cm^2 the light-induced recovery from 900 nm is significantly greater than at 810 nm which is in line with the reported triplet spectra[7] as shown in Supplementary Figure 20b. It is not possible to directly compare the absolute light-induced recovery of the 900/810 nm to the 900/592 nm one (Figure 2j). The two sets of experiments are indeed performed on a confocal (Supplementary Figure 30) and a widefield (Supplementary Figure 28) microscope, respectively. The confocal system is equipped with pulsed lasers (with respect to the CW laser of the widefield setup) and APD detectors and therefore the experiment design needs to be adjusted accordingly. To overcome the incompatibility of the absolute value we always consider the comparison of two wavelengths, namely the triplet absorption peak vs the 592 nm or the 810 nm light.

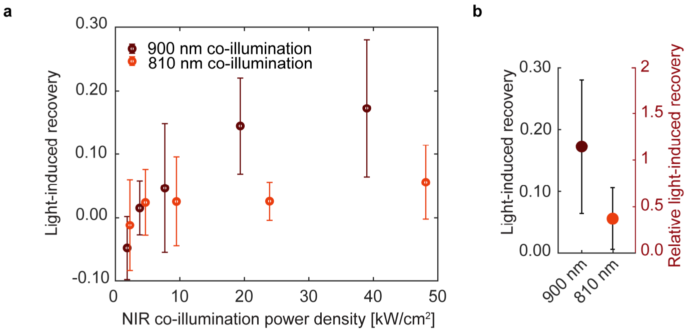

Supplementary Figure 20. (a) Light-induced recovery to NIR co-illumination. The effect of NIR co-illumination on photoswitching fatigue was studied in terms of the NIR power density (0 - 40 kW/cm^2 at 900 nm and 0 – 50 kW/cm^2 at 810 nm). The light-induced recovery was computed by comparing the normalized intensity after ~ 2000 photoswitching cycles with or without NIR co-illumination. For each condition, N ≥ 3 fatigue curves were taken. From these curves, a mean curve per condition was calculated (with its associated standard deviation, σ). The data points correspond to the difference between the mean curve at a given power density and the mean control curve after ~ 2000 photoswitching cycles. The error bars correspond to the combined error associated with both measurements. The 405 nm dose was set to 300 W/cm^2 and 1 ms while the laser was driven at 80 MHz with ~ 100 ps pulse width. The 488 nm dose was set to 14 W/cm^2 and 1 ms while the laser was driven at 80 MHz with ~ 100 ps pulse width. (b) Light-induced recovery at the maximum power density for each wavelength. The relative light-induced recovery scales with the absorption coefficients reported for the triplet state in rsEGFP2[7].

Supplementary Table 8. Exponential fit of 592 nm and 915 nm co-illumination data in the widefield microscope in Figure 2j. For each condition, N ≥ 3 fatigue curves were taken. The parameter *k* in the fitting is calculated by fitting the data to an exponential function: *a(1 – exp(PD/k))*. The 405 nm dose was set to 160 W/cm^2 for 1 ms and the 488 nm dose was set to 300 W/cm^2 and 1 ms.

Experiment	Parameters	Method	Estimated value	Confidence interval 95%
**592 nm power ramp**	***y = a(1-exp(x/k))***	***a***	0.077	0.064 – 0.091
		k	- 1.334	-2.466 – (-0.202)
**915 nm power ramp**	***y = a(1-exp(x/k))***	***a***	0.109	0.092 – 0.126
		k	- 0.420	-0.716 – (-0.123)

Supplementary Note 11: Photoswitching fatigue of other green negative photoswitchers

To test the generalizability of this mechanism in other green negative photoswitchers, we investigated the fatigue of different rsFPs as well as their response to the 592 nm co-illumination. We characterized 4 other negative photoswitchers: rsEGFP(N205S)[27], a mutant closely related to rsEGFP2[28];

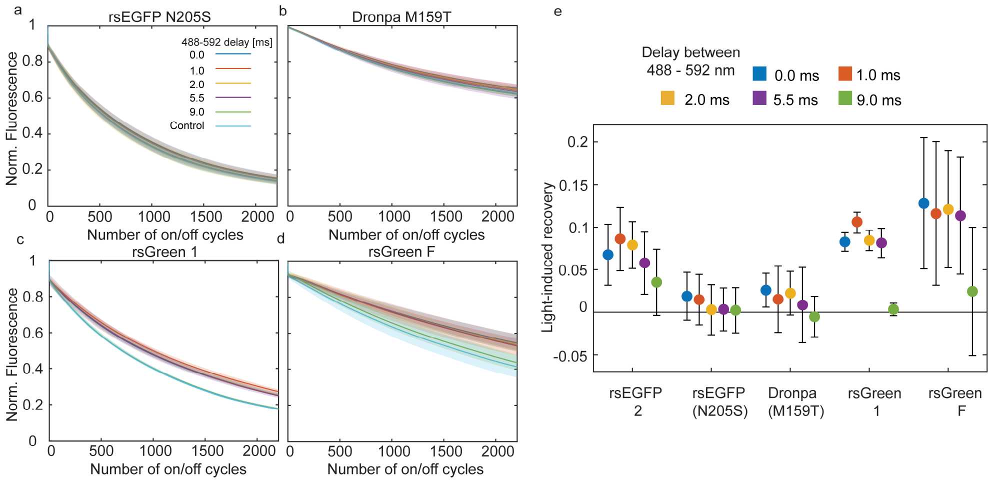

Supplementary Figure 21. (a) rsEGFP(N205S) photoswitching fatigue curve as a function of the 488-592 nm delay time. The fluorescence was normalized to the signal of first photoswitching cycle. (b) Dronpa M159T photoswitching fatigue curve as a function of the 488-592 nm delay time. The fluorescence was normalized to the signal of first photoswitching cycle. (c) rsGreen 1 photoswitching fatigue curve as a function of the 488-592 nm delay time. The fluorescence was normalized to the signal of first photoswitching cycle. (d) rsGreen F photoswitching fatigue curve as a function of the 488-592 nm delay time. The fluorescence was normalized to the signal of first photoswitching cycle. (e) Summary of the light-induced recovery, i.e. the normalized fluorescence signal recovered after 2000 on/off cycles, for the 5 RSFPs included in the study. The colour code corresponds to the delay between 488 and 592 nm pulses. For each condition, $N \geq 3$ fatigue curves were taken. From these curves a mean curve per condition was calculated (with its associated standard deviation, σ). The data points correspond to the difference between the mean curve at a given power density and the mean control curve after 2000 photoswitching cycles. The error bars correspond to the combined error associated with both measurements. The shaded areas in (a)-(d) represent ± σ associated with each mean curve for every condition.

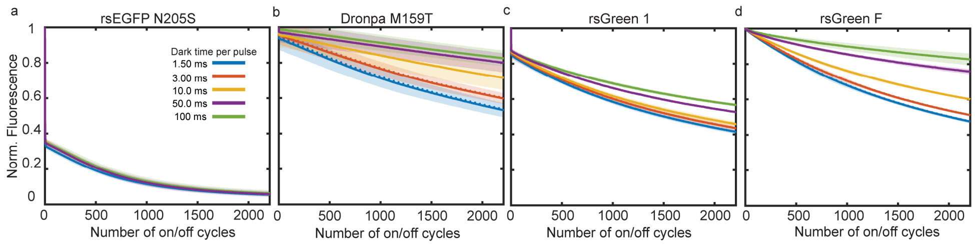

Supplementary Figure 22. (a) rsEGFP(N205S) photoswitching fatigue curve as a function of the dark waiting time. The fluorescence was normalized to the signal of first photoswitching cycle. (b) Dronpa M159T photoswitching fatigue curve as a function of the dark waiting time. The fluorescence was normalized to the signal of first photoswitching cycle. (c) rsGreen 1 photoswitching fatigue curve as a function of the dark waiting time. The fluorescence was normalized to the signal of first photoswitching cycle. (d) rsGreen F photoswitching fatigue curve as a function of the dark waiting time. The fluorescence was normalized to the signal of first photoswitching cycle. For each condition, $N \geq 3$ fatigue curves were taken. From these curves a mean curve per condition was calculated (with its associated standard deviation, σ). The shaded areas represent ± σ associated with each mean curve for every condition.

Dronpa(M159T), a green RSFP of Anthozoan origin[29]; and two variants of the rsGreen family[30], rsGreen1 and rsGreenF.

As shown in Figure 3d in the main text, the maximum recovery is observed for the rsGreen family proteins, especially notable for rsGreenF where it is > 10 % for a 592 nm power density ~ 4 kW/cm^2. In Supplementary Figure 21, we display the raw photoswitching fatigue curves from which the plot in Figure 3d was built. Each RSFP exhibits its own characteristic fatigue "fingerprint," and without detailed spectroscopic data (as we have for rsEGFP2), these differences can be hard to interpret. However, the bleaching profile of rsEGFP2 offers a useful framework. Across all proteins we tested, the slowest off-switchers, rsEGFP(N205S) and rsGreen1, always showed the fastest accumulation of fatigue. We propose that this arises from their lower off-switching quantum yields: because they require longer or more intense 488 nm illumination to reach 80–90 % off-state conversion, each cycle deposits more energy and increases the probability of populating the triplet state, which in turn accelerates bleaching.

Supporting this idea, when we introduced dark intervals after 488 nm exposure (Supplementary Figure 22), only the faster switchers recovered significantly, rsEGFP(N205S) showed no improvement even with longer wait times. Note that the minimum time camera read-out time for a 30x30 μm^2 FOV was 1.5 ms, therefore, that was set as the shortest dark time. A similar pattern emerges for the rsGreen variants: the slower rsGreen1 bleaches far more quickly than rsGreenF. In summary, slower off-switching RSFPs accumulate more triplet-state absorption per cycle, and thus more irreversible photobleaching, than their faster-switching counterparts.

Supporting Note 12: Imaging multiplexing with RSFPs at high-spatiotemporal resolution

In recent years, RSFPs have been incorporated into imaging multiplexing strategies that rely on their differentiated light-driven kinetics to unmix and recover the identity of specifically labelled structures in biological imaging. Approaches such as TMI[31] or LIGHTNING[32] carefully resolve the off-switching in time to assess the identity of the fluorophore, while strategies like exNEEMO[33,34] locate the different proteins in a multidimensional unmixing space based on their expected emission given different levels of 405 nm photoactivation. It is important to note that the better separation between different RSFPs in all these methods occurs at low excitation power densities when the characteristic off-switching time between species are more distinct. This becomes crucial in approaches which rely on a camera-based detection system – like TMI[31] and exNEEMO[33,34] – that imposes a time-resolution limit of ~ 1 ms.

To explore the working range of kinetic-based unmixing, we simulated the behaviour of 4 rsEGFP2-like RSFPs with different off-switching quantum yields (from $0.1*QY_{rsEGFP2}$ to $1*QY_{rsEGFP2}$). In particular, we track the difference in off-switching for each of these RSFPs compared with the prototypical rsEGFP2 as a function of the off-switching power density, i.e. 488 nm. For each simulated fluorophore, the off-switching curve was simulated (with 2% Gaussian noise added to mimic experimental conditions) and fitted with a monoexponential decay function to extract the characteristic time, $\tau_{fluorophore}$. The $\tau_{fluorophore}$ of each simulated fluorophore was subtracted to the extracted $\tau_{rsEGFP2}$ at the same 488 nm power density to obtain the $\Delta\tau$ between each simulated RSFP and rsEGFP2. The results of the simulations are shown in Supplementary Figure 23, below. At lower power densities (< 100 W/cm^2) the separation between species is larger than the time-resolution of the camera (orange box in Supplementary Figure 23) making it possible to distinguish them from rsEGFP2, however, as we approach the typical 488 nm power densities for high-spatiotemporal resolution imaging (green-shaded area, 0.1 – 2 kW/cm^2) the difference between the characteristic times shrinks and we are rapidly below the camera time-resolution limit cut-off (red-shaded area, < 1 ms). For methods that depend on resolving in time the off-switching kinetics of different probes simultaneously such as TMI[31], the number of fluorophores that can be unmixed at power densities for high-spatiotemporal resolution imaging is therefore limited by the camera read-out time.

Other methods such as exNEEMO[33,34] while not relying on resolving the off-switching process can see a decrease in the accuracy of the unmixing since the fluorescence emission of negative photoswitchers

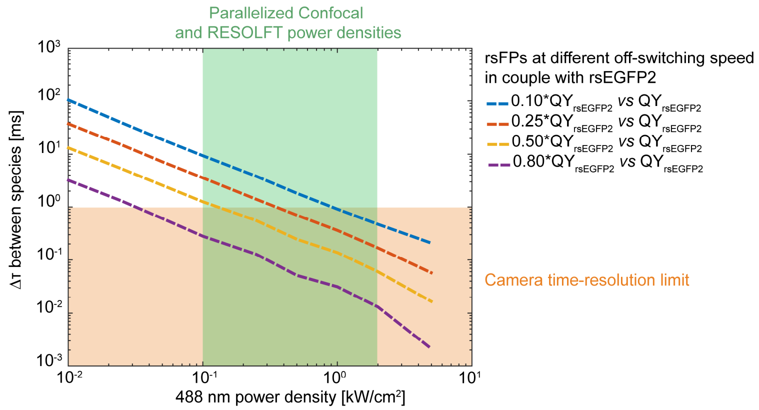

Supplementary Figure 23. Difference between off-switching characteristic times as a function of the 488 nm power density. The off-switching of 5 different rsEGFP2-like fluorophores was simulated at multiple power densities with 2% Gaussian noise. Each RSFP has a different off-switching quantum yield (from $0.1*QYrsEGFP2$ to $1*QYrsEGFP2$) and the characteristic off-switching time, $\tau_{fluorophore}$, is extracted by fitting a monoexponential decay function. Each $\tau_{fluorophore}$ is compared to τrsEGFP2, to compute the difference $\Delta\tau$ in ms. In the green-shaded area, we show the range of power densities used typically in high-spatiotemporal resolution imaging. When $\Delta\tau$ falls below the camera time-resolution cut-off, the proteins are not easily distinguishable only relying on the camera's time-resolution.

is linked to the off-switching kinetics. If two RSFPs only differ in their off-switching quantum yields, such as in the simulation of Supplementary Figure 23, their relative positions in the unmixing space will be determined by the integral below the off-switching curve. Therefore, if the off-switching process of both RSFPs is similar enough (and all other spectroscopic properties are equal between the RSFPs) so it will be the emitted fluorescence.

The green shaded area in Figure 23 highlights the typical power densities used in parallelized confocal or RESOLFT with RSFPs. Although these techniques deliver high spatio–temporal resolution, they operate under conditions where the kinetic differences between RSFPs approach, or fall below, the limits of what conventional multiplexing strategies can reliably resolve. Identifying additional dimensions for unmixing (i.e. bleaching profile) is crucial to solve the ambiguities that naturally occur at such power densities.

Supplementary Note 13: Photobleaching recovery with 592 nm co-illumination in live-cell imaging

Aiming to benchmark the measured photobleaching and recovery, we imaged a variety of samples containing rsEGFP2 in parallelized confocal mode and quantified the observed loss of fluorescence with and without 592 nm co-illumination. We set the imaging conditions to maximize the temporal resolution of the acquisition with 4 ms total dwell time and 60 nm scanning steps and monitored the fluorescence over time for 50 imaging frames. As shown in Supplementary Figure 24a, different live-cell samples (bacteria, vimentin, actin and mitochondria) have their characteristic bleaching curve, additionally, we incorporated a sample of rsEGFP2 embedded in a PAA matrix at the same concentration as in the characterization experiments. We observed photobleaching between 20 to 40 % in eukaryotic cells, with actin-labelled rsEGFP2 showing higher bleaching resistance (29% normalized fluorescence at the last frame in magenta) compared to rsEGFP2 tagged to vimentin and mitochondria (15% and 16% normalized fluorescence at the last frame, in yellow and green, respectively). In prokaryotic cells, we observed the highest photobleaching resistance (52% normalized fluorescence at the last frame in orange) which we attributed to the specific geometry of the sample, i.e. a thick pellet of bacterial cells rather than a semi-2D sample like for the other samples. Similarly, we measured the photobleaching with 592 nm co-illumination (15 kW/cm^2 and 0.5 ms) added in the pulse scheme and we observed a decrease in photobleaching in all samples, specifically higher in bacteria (21% recovery in orange) and in the PAA layer (13% in blue). An interpretation of these results can be linked to a slower diffusivity of molecular oxygen both in biofilms[35,36] and in the PAA matrix[37–39] which can prolong the lifetime of the triplet and reduce its reactivity downstream to generate irreversible photobleaching. In this context, given that live-cells are imaged in aqueous media, is plausible that the reactivity of the triplet state of rsEGFP2 generated more photoproducts that can lead to photobleaching and the observed fluorescence loss.

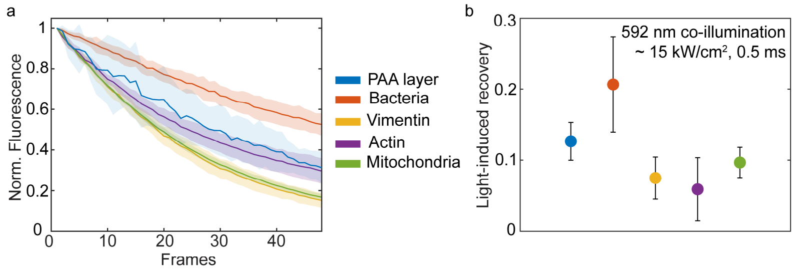

Supplementary Figure 24. (a) Intensity traces for a variety of samples containing rsEGFP2 imaged in parallelized confocal mode. In blue, a rsEGFP2-embedded polyacrylamide layer; in orange, a biofilm of *Escherichia Coli* expressing rsEGFP2; in yellow, rsEGFP2-vimentin in U2OS cells; in magenta, rsEGFP2-ActinChromobody in U2OS cells, and in green, rsEGFP2-OMP2 in U2OS cells. The lines represent the mean intensity traces and the shaded area shows ± σ from the mean. (b) Light-induced photobleaching recovery with 592 nm co-illumination in the aforementioned samples. The 592 nm illumination dose was 0.5 ms at 15 kW/cm^2. The dots represent the mean light-induced recovery calculated from the difference between the mean fluorescence loss without and with 592 nm light and the error bars show ± σ from the mean calculated by propagating the error from the two experimental conditions.

To monitor the photobleaching experienced in live-cells expressing rsEGFP2-OMP25 in parallelized confocal mode, for every image, the integrated intensity of 4 regions of interest (ROIs) was calculated – 1 of the ROIs was used to measure the image background and the remaining 3 ROIs contained the labelled structure – and monitored across the 500 frames of the time-lapse recordings. In Supplementary Figure 25, we show the normalized average fluorescence decay (25a) and raw average fluorescence decay (25b) for the images shown in Figure 4a in the main text. The intensity trace when the 592 nm is applied to the imaging pulse scheme decays slower than without 592 nm co-illumination. As seen in

Supplementary Figure 25b both images presented similar levels of initial intensity, however, applying the 592 nm co-illumination improves the photobleaching resistance in that pool of mitochondria.

From the intensity traces, we obtained the photobleaching profiles for each image which were fitted with a monoexponentially decaying function, and as a result, we obtained the bleaching constant for every image. In Supplementary Figure 25c, we show the bleaching constant for every image as a function of their signal-to-background ratio in the first frame of the acquisition. We observed that adding the 592 nm co-illumination slows down the photobleaching experienced by the mitochondria (larger bleaching constants in Supplementary Figure 25a). In that sense, the 592 nm co-illumination prolonged the time-lapse by tenths of frames in recordings showing similar initial signal-to-background ratios proving to be a powerful method for minimizing photobleaching in samples where the brightness of the cellular structures of interest is limited by the transfection efficiency.

We further tested the effect of the 592 nm co-illumination in the observed photobleaching of live-cells in a super-resolution imaging context, specifically, RESOLFT microscopy within the MoNaLISA modality. We evaluated the photobleaching recovery effect by comparing the normalised fluorescence signal after 20 imaging frames with and without the secondary illumination in vimentin and actin endogenously tagged with a set of rsFPs. To build the respective bleaching curves, the signal from two sub-areas – one corresponding to the structure, one corresponding to the dark background, each of ~ 8×8 μm^2 – of the MoNaLISA FOV (~ 40×40 μm^2) was monitored across the time-lapse recording. The counts per area of each region are calculated and the background is subtracted on a frame-by-frame

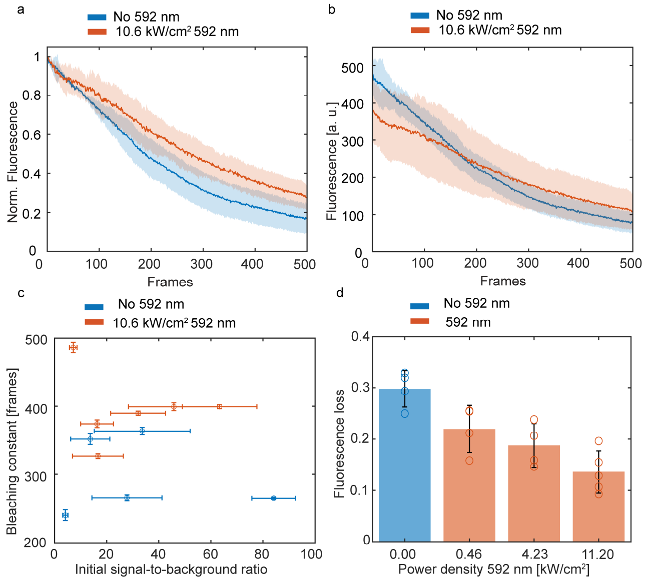

Supplementary Figure 25. (a) Normalized fluorescence trace of example images in Figure 4a in the main text. Adding 592 nm co-illumination decreases the photobleaching rate. The shaded area represents the standard deviation of the mean intensity from the ROI where the signal is measured. (b) Raw fluorescence trace of example images in Figure 4a in the main text. Adding 592 nm co-illumination decreases the photobleaching rate. The shaded area represents the standard deviation of the mean intensity from the ROI where the signal is measured. (c) Bleaching constant as a function of the initial signal-to-background ratio. A larger bleaching constant indicates slower bleaching. At a similar initial signal-to-background ratio, the addition of 592 nm co-illumination extends the available number of frames. (d) Bleached fraction after 20 MoNaLISA frames for different 592 nm illumination power densities in vimentin tagged with rsEGFP2 in U2OS cells. The bleached fraction is reduced as the 592 nm power density increases. The bars show the mean fluorescence loss after 20 MoNaLISA imaging frames ± σ. Overlayed, the fluorescence loss calculated from individual cells for all conditions.

basis, afterwards, the signal is normalised to the first frame. The results are extracted from an average of 3-5 cells (or curves) per condition.

As we observed in the PAA gels for rsEGFP2 the recovery effect is greater when a large share of the protein's ensemble population has undergone both 405 and 488 nm excitations, therefore, we added the 592 nm illumination to the 488 nm patterned illumination path. With this optical design, we made sure that the 592 nm multi-foci pattern was co-aligned to both 405 and 488 nm multi-foci patterns. We tested the recovery effect from the 592 nm co-illumination in vimentin labelled with rsEGFP2 in U2OS cells as a function of the irradiation power density with an analysis pipeline as described above. The results displayed in Supplementary Figure 25b show a decrease in the bleached fraction after 20 imaging frames as the 592 nm illumination power density increases.

Additionally, we characterized the SNR and spatial resolution at different time points within a timelapse recording from U2OS cells tagged with LifeAct-rsEGFP2 shown in Figure 4c in the main text. We traced ~ 20 – 40 line profiles of actin filaments per image and performed a Lorentzian fitting with a custom-written MATLAB script. We calculated the SNR as well as the FWHM of the Lorentzian peak of each line profile in the first and last frame of each timelapse recording. As shown in Supplementary Figure 26a, the SNR with or without 592 nm co-illumination is higher in the initial frame (median values 14.01 with 592 nm, 10.88 without), however we observed a greater reduction in SNR without 592 nm co-illumination (~ 40 % decrease in SNR after 20 RESOLFT imaging frames) than when the 592 nm co-illumination is applied (~ 27 % decrease in SNR after 20 RESOLFT imaging frames). Along with the intensity traces shown in Figure 4c of the main text, the SNR quantification highlights the benefits of incorporating the 592 nm co-illumination for prolonged timelapse imaging in intensity-demanding techniques such as MoNaLISA. At the same time, we quantified the FWHM measured on > 100 actin filaments per condition in Supplementary Figure 26b, and, as expected, there is a loss in resolution in the last imaging frame with and without 592 nm co-illumination. Although the median values for all conditions are similar, the data reflect that the distribution of sizes of the filaments in the 20th frame without 592 nm co-illumination is skewed towards broader filaments, indicating a loss in resolution (Supplementary Figure 26b 20th frame blue). As a comparison, that does not seem to happen when the 592 nm co-illumination is applied where the distribution of the filament sizes is comparable in the 1st and 20th frames (Supplementary Figure 26b 1st and 20th frames orange). Similarly, we quantified the number of fitted FWHM that fell within 3σ of the median and compared it to the number

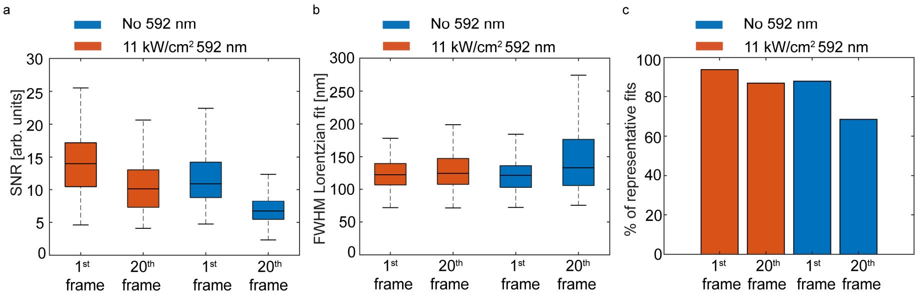

Supplementary Figure 26. (a) SNR of U2OS-LifeAct-rsEGFP2 measured from > 100 actin filaments per condition. The size of the box represents the 25th-75th percentiles of the data, and the lines represent the median value. The whiskers extend to 1.5 x the interquartile distance below or above the 25th-75th percentiles. The reduction in SNR at the 20th frame is less significant when 592 nm co-illumination is applied (27 %) than when is not (40 %). (b) FWHM from Lorentzian fit of U2OS-LifeAct-rsEGFP2 measured from > 100 actin filaments per condition. The size of the box represents the 25th-75th percentiles of the data, and the lines represent the median value. The whiskers extend to 1.5 x the interquartile distance below or above the 25th-75th percentiles. The distribution of sizes of the measured filaments in the 20th frame without 592 nm co-illumination is skewed towards broader filaments indicating a loss of resolution. The number of filaments analysed for each condition, from left to right, N=139, N=133, N=124, N=105. (c) Percentage of fits that lie within 3σ of the median. Applying the 592 nm co-illumination increases the number of representative fits at the 20th frame.

of line profiles input to the fitting algorithm (Supplementary Figure 26c). Interestingly, we see a decrease in the number of quality fits in the last frame of the recording when the 592 nm co-illumination is not applied, moreover, that decrease is not that significant when adding the 592 nm co-illumination in the pulse scheme suggesting that more filaments are still visible and their sizes retrievable with better accuracy after 20 MoNaLISA imaging frames.

Supplementary Note 14: Phototoxicity assessment in live-cell imaging context

To assess if the 592 nm co-illumination led to any adverse effects in the imaged cells, we monitored the behaviour of mitochondria under parallelized confocal illumination conditions. In particular, we manually annotated the occurrence of events that report on the mitochondrial network mobility. Such events include branching, visualized as transient deformations of the mitochondrial membrane; fission; fusion and long stretching/retraction, which appear as transient and pronounced elongation or retraction of the mitochondrial tubules. Examples of such events are shown in Supplementary Figure 27a. The frame number when the events occurred was noted down as a timestamp for all the observed events in all the images with and without 592 nm co-illumination and summed together in bins of 25 frames. In total, we observed 224 such events in 5 images without 592 nm and 321 events in 6 images with 592 nm co-illumination. From these pool events, the cumulative probability distribution was computed as shown in Supplementary Figure 27b. Our data suggest that the addition of the 592 nm co-illumination did not affect the mobility of the mitochondrial network as represented by the counted events as both datasets, with and without 592 nm, show a similar distribution across time. Moreover, we did not see changes in the mitochondrial network morphology typically associated with phototoxicity such as swelling or blobbing.

Additionally, we investigated whether the 592 nm co-illumination had adverse effects on the cells that were being imaged. For that purpose, we used the DNA repairing protein XRCC1 (X-Ray cross complementary factor 1)[40] as a quantitative reporter for light-induced damage in the cell's nucleus. XRCC1 acts as a central loading platform for DNA repair[40] and it manifests as a bright puncta in the nuclei. In our photodamage assay, we counted the bright puncta in the nucleus of different cells (N = 8-10) before and after taking a MoNaLISA image with different 592 nm co-illumination power densities.

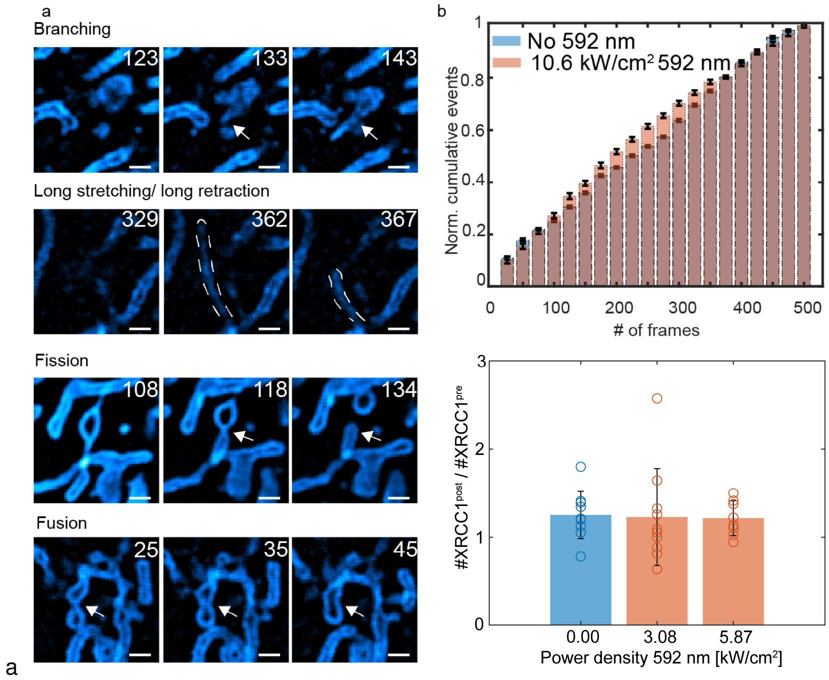

Supplementary Figure 27. (a) Examples of reported events in the mitochondria datasets. The scale bars are 1 µm. (b) Cumulative probability distribution of the observed events across the 500 frames of the parallelized confocal recording. In total, 224 events were annotated in 5 images without 592 nm co-illumination and 321 events in 6 images with 592 nm co-illumination. (c) Light-induced DNA damage assay. The bright puncta on the cell nucleus from DNA damage reporter XRCC1 were counted before and after taking a MoNaLISA image with different 592 nm power densities. The addition of the 592 nm illumination didn't lead to an increase in light-induced DNA damage. The bars show the average number of puncta from the XRCC1 reporter after illumination compared to pre-illumination. Overlayed, the ratio of puncta for each individual cell.

As shown in Figure 27c, the additional 592 nm illumination did not incur an increase in light-induced DNA damage compared to a normal MoNaLISA imaging recording.

Supplementary Note 15: Optical set-ups

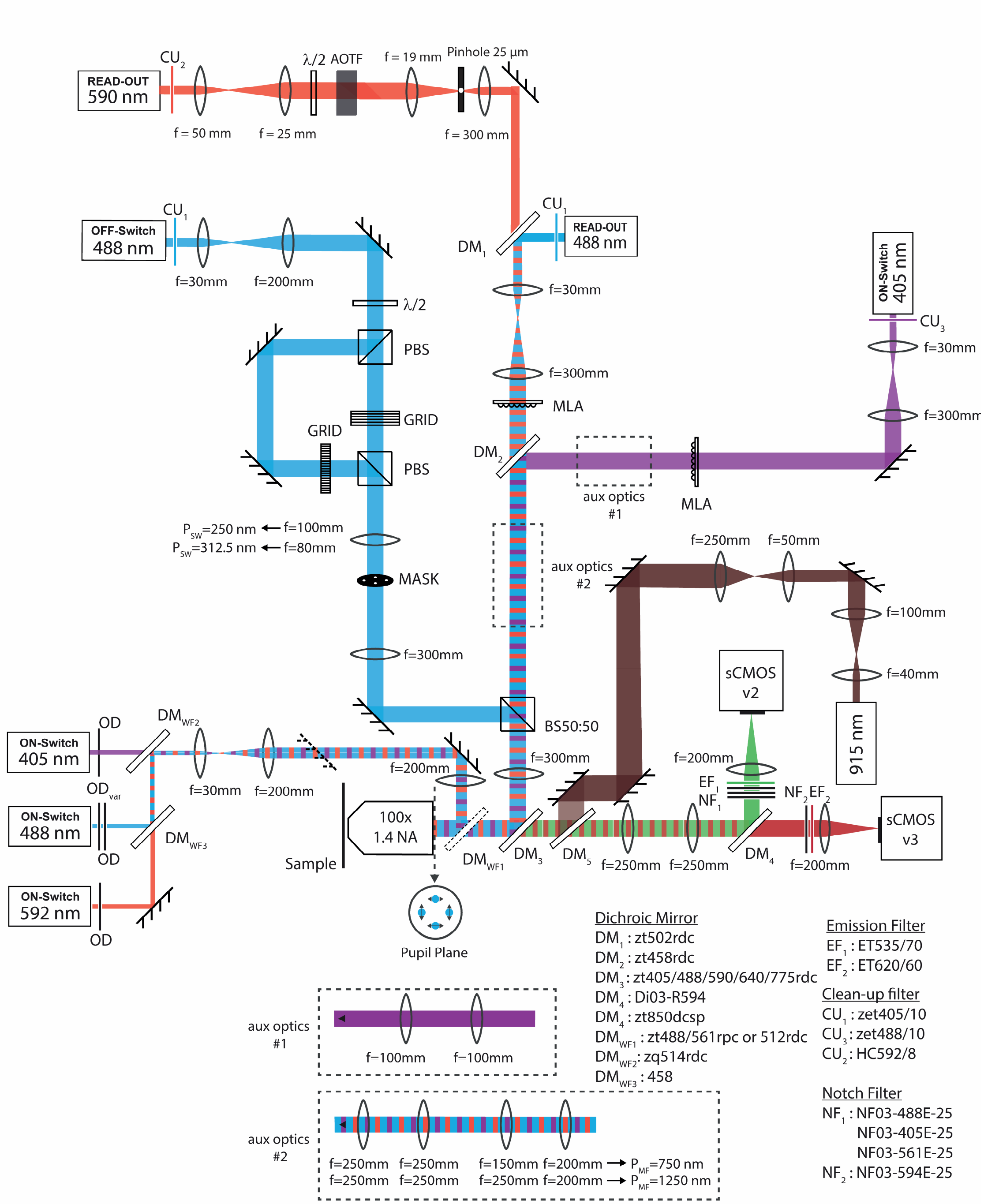

Supplementary Figure 28. Widefield (WF) microscope set-up. The photoswitching fatigue of all proteins was carried out on this microscope as well as the RESOLFT imaging. Two pairs of microlenses array (MLA) are incorporated in the optical paths to create the multi-foci pattern at the sample plane. These optical elements were removed from the set-up when using the WF mode for photoswitching fatigue characterization. The off-switching path was only used in imaging to create the confinement necessary to achieve sub-diffractive resolution.

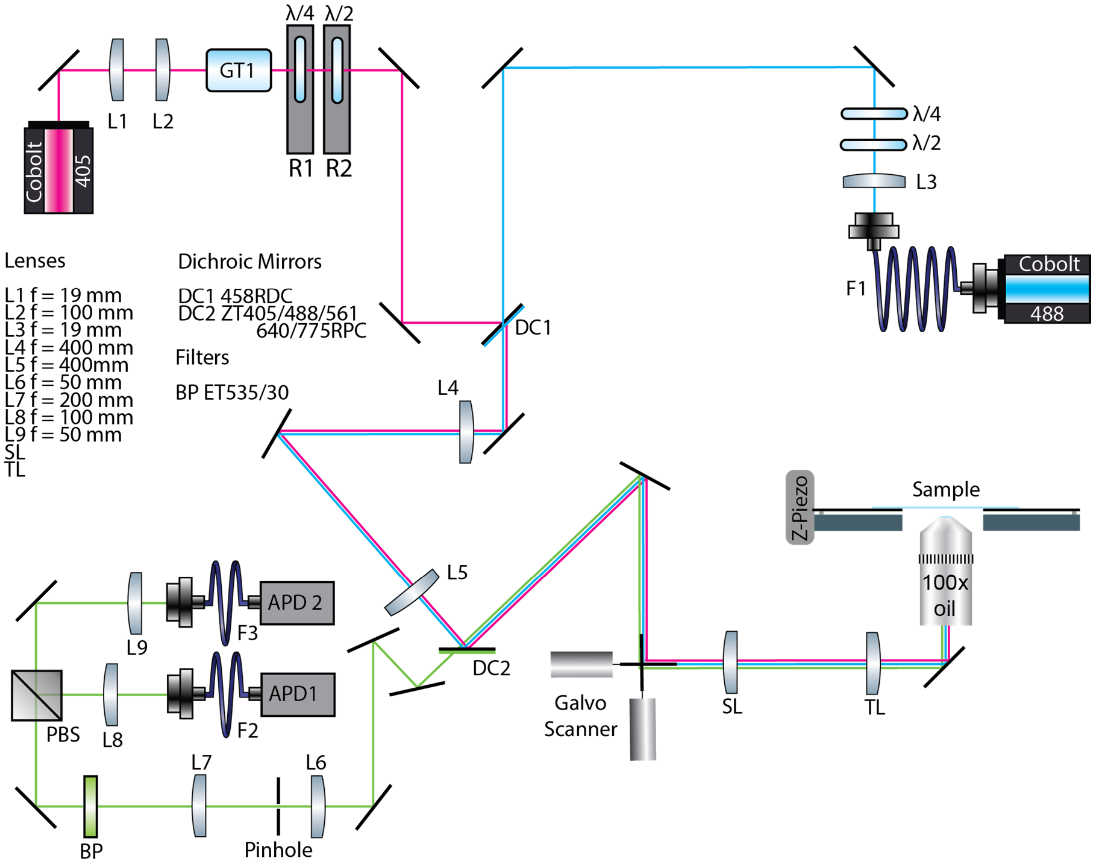

Supplementary Figure 29. Point-scanning microscope set-up. This microscope was employed for the characterization experiments that required higher temporal resolution. The microscope incorporates two point-detectors with high temporal resolution. The signal from both detectors was summed to create the final signal. Elements not listed in the Figure are listed below. Fiber Optics: F2: FG105LGA (Thorlabs), F3: AFS50 (Thorlabs). Polarization rotators: R1 and R2: K10CR1/M (Thorlabs), Polarization Optics: GT1: GTH10M-A (Thorlabs), λ/4: 460-680 achr. (B. Halle Nachfl, Berlin, Germany), λ/2: 460-680 achr. (B. Halle Nachfl), PBS: CCM2- PBS251/M (Thorlabs). Lenses: SL: 50 mm (Leica Microsystems, Wetzlar, Germany), TL: 200 mm (Leica). Scanners: Galvo Scanner XY: 6215H Galvanometric mirrors + 71215HHJ Servo Driver (Cambridge Technology, Bedford, MA, USA), Piezo Stage Z: LT-Z-100 (Piezoconcept, Lyon, France).

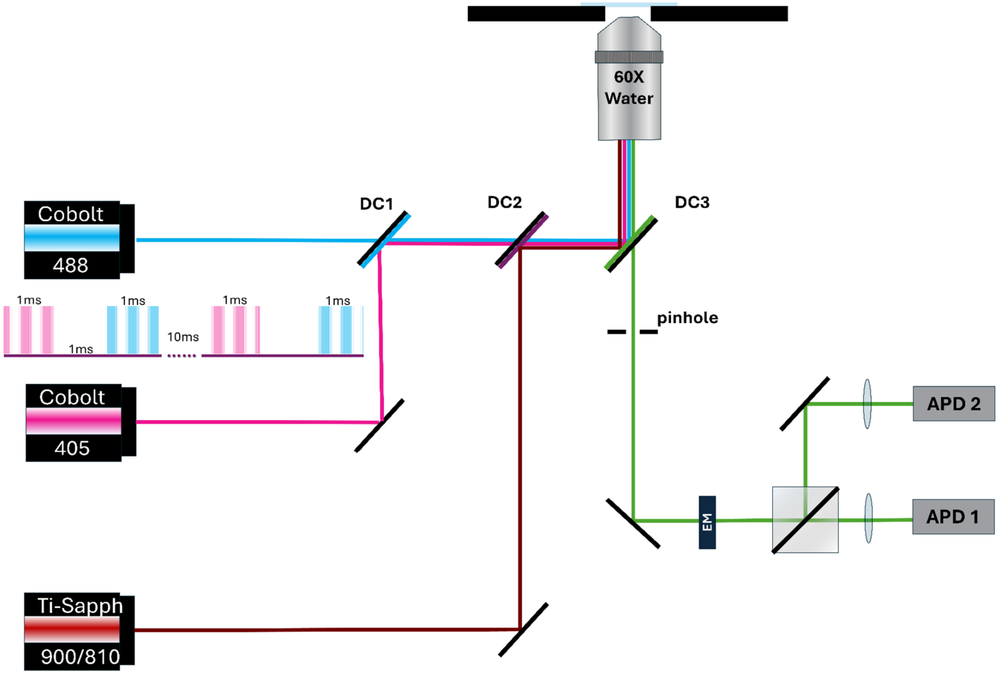

Supplementary Figure 30. Near-infrared confocal microscope set-up. This microscope was employed for the characterisation experiments in the NIR region. The longer wavelength co-illumination was tuned employing the Ti: Sapphire laser. The microscope incorporates two point-detectors with high temporal resolution. The signal from both detectors was summed to create the final signal.

Supplementary Note 16: Photophysical simulation of rsEGFP2

To characterize the behaviour observed in the photoswitching experiments we developed a kinetics simulation tool that computes the time-evolution of the emissive state concentration according to a network of interconnected electronic states. The simulation tool was developed as Python script and solves analytically the system of linear ordinary differential equations that describe the photophysics of the fluorophore. The theoretical framework is briefly described in this note.

Being C_i the concentration of species i, the general set of rate equations for a system having j such species will be defined as

$$\frac{dC_i}{dt} = \sum_j k_{ij} C_j \tag{1}$$

where k_{ij} is the kinetic constant for the reaction that transforms the *j-th* species into the i species when $j \neq i$. The kinetic constant k_{ii}, represents the depopulation of the i species according to a kinetic scheme. It is calculated by summing all the kinetic rates of transitions reducing the concentration of i

$$k_{ii} = -\sum_{j \neq i} k_{ji} \tag{2}$$

In general, to describe the concentration evolution of state i within a network of j interconnected states one must solve a set of differential equations such as (1) for each of the involved species. In that regard, the problem can be expressed in matrix form and simplifies to

$$\frac{d\boldsymbol{C}(t)}{dt} = \boldsymbol{KC} \tag{3}$$

All the kinetic rates connecting states in the system are collected in matrix **K** in equation 3. In that sense, the constant describing the $i \rightarrow j$ reaction will be indexed in position k_{ji}. Similarly, vector **C** contains the concentrations for each state with the same indexing as **K**. The solution of the differential equation in the matrix form is analogous to its scalar equation and yields,

$$\boldsymbol{C}(t) = \exp(\boldsymbol{K}t)\, \boldsymbol{C}(t = 0) \tag{4}$$

Being **C** $(t = 0)$ the set of initial concentrations for each species. From here, calculating the matrix exponential requires solving an eigenvalue problem[41], since

$$\exp(\boldsymbol{K}t) = \boldsymbol{U}\exp(\boldsymbol{\lambda}t)\boldsymbol{U^{-1}}, \qquad \text{with } \boldsymbol{K} = \boldsymbol{U\lambda U^{-1}} \tag{5}$$

where **λ** is the diagonal eigenvalues matrix and **U** is the eigenvectors matrix. Therefore, to obtain the concentration at any time point for a given fluorophore one should construct a suitable kinetic matrix **K** with its photophysical parameters and consider a reasonable set of initial conditions.

Generally, the kinetic constants connecting the different species within the scheme will represent spontaneous/thermally induced processes (non-light-induced) or light-induced processes. Since the probability of the latter is modulated by the irradiation intensity, the kinetic matrix needs to include the photon fluxes for each illumination dose within the pulse scheme. To practically emulate any experiment, we represent the experimental pulse scheme as a concatenation of so-called kinetic windows. For every kinetic window, the status of all the illumination wavelengths in the pulse scheme is defined – on or off –, as well as the photon flux delivered to the sample during that time. The different

lasers are simulated as square waves, and two simplified detection modes are implemented: time-resolved or integrated signal.

Supplementary Table 9. Parameters used in the characterization experiments with the different rsFPs.

Experiment	405 nm dose	488 nm dose	592 nm dose
**Photoswitching fatigue characterization (Supplementary Figure 1 b,c,d,e)**	1 ms	1 ms	-
	~ 0.01 – 0.5 kW/cm^2	~ 0.03 – 2 kW/cm^2	-
**On-Switching curve (Supplementary Figure 6)**	1 ms	2.0 ms	-
	0 - ~ 0.65 kW/cm^2	120 W/cm^2	-
**592 nm power ramp, low 488 power (Figure 2f, Supplementary Figure 16a)**	1.0 ms	1.5 ms	1.0 ms
	230 W/cm^2	200 W/cm^2	0 – 7.4 kW/cm^2
**592 nm power ramp, high 488 power (Figure 2f, Supplementary Figure 16b)**	1.0 ms	0.9 ms	1.0 ms
	200 W/cm^2	420 W/cm^2	0 – 7.4 kW/cm^2
**488 – 592 nm delays rsEGFP2 (Figure 2g, Supplementary Figure 17)**	1.0 ms	1.0 ms	1.0 ms
	170 W/cm^2	250 W/cm^2	7.0 kW/cm^2
**Photoswitching fatigue characterization (Figure 1b-d,f, Supplementary Figure 8a,b)**	1 ms	2.5 – 0.5 ms	-
	10 – 450 W/cm^2	70 -930 W/cm^2	-
**Thermal recovery rsEGFP2 (Figure 1g, Supplementary Figure 11, 19a)**	1 ms	5 ms	-
	240 W/cm^2	40 W/cm^2	-
**Confocal 405 – 488 nm delays (Supplementary Figure 5a,b)**	5 µs	500 µs	-
	30 kW/cm^2	40 kW/cm^2	-
**Confocal 488 – 488 nm delays (Figure 2b, Supplementary Figure 15)**	5 µs	500 µs	-
	30 kW/cm^2	40 kW/cm^2	-
**On-switching doses with constant total energy rsEGFP2 (Figure 2a)**	20 – 0.3 ms	1 ms	-
	3 – 200 W/cm^2	200 W/cm^2	-
**488 – 592 nm delays rsEGFP(N205S) (Figure 3a,d, Supplementary Figure 21a)**	1 ms	3.7 ms	1 ms
	160 W/cm^2	200 W/cm^2	4.16 kW/cm^2
**488 – 592 nm delays Dronpa(M159T) (Figure 3a,d, Supplementary Figure 21b)**	1 ms	0.6 ms	1 ms
	160 W/cm^2	220 W/cm^2	4.00 kW/cm^2
**488 – 592 nm delays rsGreen1 (Figure 3a,d, Supplementary Figure 21c)**	1 ms	1.5 ms	1 ms
	160 W/cm^2	190 W/cm^2	3.75 kW/cm^2
**488 – 592 nm delays rsGreenF (Figure 3a,d, Supplementary Figure 21d)**	1 ms	1.0 ms	1 ms
	160 W/cm^2	240 W/cm^2	4.27 kW/cm^2
**Thermal recovery rsEGFP(N205S) (Supplementary Figure 22a)**	1 ms	1.5 ms	-
	7 W/cm^2	2.4 kW/cm^2	-
**Thermal recovery Dronpa(M159T) (Supplementary Figure 22b)**	1 ms	0.6 ms	-
	80 W/cm^2	220 W/cm^2	-
**Thermal recovery rsGreen1 (Supplementary Figure 22c)**	1 ms	2.0 ms	-
	240 W/cm^2	110 W/cm^2	-
**Thermal recovery rsGreenF (Supplementary Figure 22d)**	1 ms	1.0 ms	-
	240 W/cm^2	240 W/cm^2	-

**NIR power ramp (Figure 2g, Supplementary Figure 20)**	80 MHz for 1 ms	80 MHz for 1 ms	~ 50 s
	300 W/cm^2	14 kW/cm^2	0 – 50 kW/cm^2
**592 nm vs 915 nm power ramps (Figure 2j, Supplementary Table 9)**	1.0 ms	1.0 ms	1 ms
	170 W/cm^2	250 W/cm^2	592 nm 0 - ~ 15 kW/cm^2<br>915 nm 0 - ~ 4 kW/cm^2

Supplementary Table 10. Parameters used in the imaging experiments with the different rsFPs.

Experiment	405 nm dose	488 nm dose	592 nm dose
**Photoswitching fatigue in vimentin-rsEGFP2 in U2OS cells (Supplementary Figure 25d)**	0.5 ms	1.7 ms Off-Switching<br>1 ms Read-out	1.0 ms
	200 W/cm^2	1.2 kW/cm^2 Off-Switching<br>0.5 kW/cm^2 Read-out	0 – 11.2 kW/cm^2-
**Photoswitching fatigue in LifeAct-rsEGFP2 in U2OS cells (Figure 4c,d, Supplementary Figure 25)**	0.5 ms	1.7 ms Off-Switching<br>1 ms Read-out	1.0 ms
	200 W/cm^2	1.2 kW/cm^2 Off-Switching<br>0.5 kW/cm^2 Read-out	0 & 11 kW/cm^2
**Photoswitching fatigue in LifeAct-rsEGFP(N205S) in U2OS cells (Figure 4d)**	0.5 ms	4 ms Off-Switching<br>1 ms Read-out	1.0 ms
	75 W/cm^2	1.12 kW/cm^2 Off-Switching<br>0.71 kW/cm^2 Read-out	4.8 kW/cm^2
**Photoswitching fatigue in LifeAct-Dronpa(M159T) in U2OS cells (Figure 4d)**	0.5 ms	1 ms Off-Switching<br>1 ms Read-out	1.0 ms
	120 W/cm^2	1.12 kW/cm^2 Off-Switching<br>0.71 kW/cm^2 Read-out	6.5 kW/cm^2
**Photoswitching fatigue in LifeAct-rsGreen1 in U2OS cells (Figure 4d)**	0.5 ms	1.3 ms Off-Switching<br>1 ms Read-out	1.0 ms
	120 W/cm^2	1.2 kW/cm^2 Off-Switching<br>0.71 kW/cm^2 Read-out	4.8 kW/cm^2
**Photoswitching fatigue in LifeAct-rsGreenF in U2OS cells (Figure 4d)**	0.5 ms	1.3 ms Off-Switching<br>1 ms Read-out	1.0 ms
	75 W/cm^2	1.12 kW/cm^2 Off-Switching<br>0.71 kW/cm^2 Read-out	4.8 kW/cm^2
**DNA damage assay (Supplementary Figure 27c)**	0.5 ms	1.7 ms Off-Switching<br>1 ms Read-out	1.0 ms
	200 W/cm^2	1.2 kW/cm^2 Off-Switching<br>0.5 kW/cm^2 Read-out	0 - ~ 6.0 kW/cm^2-
**Photoswitching fatigue in OMP-25-rsEGFP2 in U2OS cells (Figure 4a, Supplementary Figure 25a,b,c, Supplementary Figure 26a,b)**	0.5 ms	1 ms	1.0 ms
	100 W/cm^2	0.5 kW/cm^2 Read-out	0 & 10.6 kW/cm^2
**Multiplexing of 4 RSFPs in parallelized confocal (Figure 3b,c)**	0.5 ms / 3 ms	5 ms	-
	100 W/cm^2	0.9 kW/cm^2	-
**Unmixing with WF + Imaging in parallelized confocal (Figure 4b)**	0.5 ms	5 ms	1.0 ms
	350 W/cm^2	0.9 kW/cm^2	10.6 kW/cm^2

**On-switching doses in cells (Figure 2b, Supplementary Figure 14)**	20 – 0.25 ms	1 ms	-
	14 W/cm^2 – 1.06 kW/cm^2	560 kW/cm^2 Read-out	-
**Bleaching in different samples (Supplementary Figure 24)**	0.5 ms	0.5 ms	0.5 ms
	1.7 kW/cm^2	0.8 kW/cm^2	15.5 kW/cm^2

References

1. Woodhouse, J. *et al.* Photoswitching mechanism of a fluorescent protein revealed by time-resolved crystallography and transient absorption spectroscopy. *Nat. Commun.* **11**, 741 (2020).
2. Uriarte, L. M. *et al.* Structural Information about the *trans* -to- *cis* Isomerization Mechanism of the Photoswitchable Fluorescent Protein rsEGFP2 Revealed by Multiscale Infrared Transient Absorption. *J. Phys. Chem. Lett.* **13**, 1194–1202 (2022).
3. Coquelle, N. *et al.* Chromophore twisting in the excited state of a photoswitchable fluorescent protein captured by time-resolved serial femtosecond crystallography. *Nat. Chem.* **10**, 31–37 (2018).
4. Nienhaus, K. & Nienhaus, G. U. Chromophore photophysics and dynamics in fluorescent proteins of the GFP family. *J. Phys. Condens. Matter* **28**, 443001 (2016).
5. Volpato, A. *et al.* Extending fluorescence anisotropy to large complexes using reversibly switchable proteins. *Nat. Biotechnol.* **41**, 552–559 (2023).
6. El Khatib, M., Martins, A., Bourgeois, D., Colletier, J.-P. & Adam, V. Rational design of ultrastable and reversibly photoswitchable fluorescent proteins for super-resolution imaging of the bacterial periplasm. *Sci. Rep.* **6**, 18459 (2016).
7. Rane, L. *et al.* Light-Induced Forward and Reverse Intersystem Crossing in Green Fluorescent Proteins at Cryogenic Temperatures. *J. Phys. Chem. B* **127**, 5046–5054 (2023).
8. Byrdin, M., Duan, C., Bourgeois, D. & Brettel, K. A Long-Lived Triplet State Is the Entrance Gateway to Oxidative Photochemistry in Green Fluorescent Proteins. *J. Am. Chem. Soc.* **140**, 2897–2905 (2018).
9. Grotjohann, T. *et al.* rsEGFP2 enables fast RESOLFT nanoscopy of living cells. *eLife* **1**, e00248 (2012).
10. Liaros, N. *et al.* Elucidating complex triplet-state dynamics in the model system isopropylthioxanthone. *iScience* **25**, 103600 (2022).
11. Ludvikova, L. *et al.* Near-infrared co-illumination of fluorescent proteins reduces photobleaching and phototoxicity. *Nat. Biotechnol.* (2023) doi:10.1038/s41587-023-01893-7.
12. Byrdin, M. & Byrdina, S. Impact of triplet state population on GFP-type fluorescence and photobleaching. *Biol. Cell* **117**, e2400076 (2025).
13. Peng, B. *et al.* Optically Modulated and Optically Activated Delayed Fluorescent Proteins through Dark State Engineering. *J. Phys. Chem. B* **125**, 5200–5209 (2021).
14. Lu, Y.-H. *et al.* Sequential Two-Photon Delayed Fluorescence Anisotropy for Macromolecular Size Determination. *J. Phys. Chem. B* **127**, 3861–3869 (2023).
15. Tkachenko, N. V. *Optical Spectroscopy: Methods and Instrumentations*. (Elsevier Science, 2006).
16. Testa, I., D'Este, E., Urban, N. T., Balzarotti, F. & Hell, S. W. Dual Channel RESOLFT Nanoscopy by Using Fluorescent State Kinetics. *Nano Lett.* **15**, 103–106 (2015).
17. Klán, P. & Wirz, J. Photochemistry of Organic Compounds: From Concepts to Practice. in (2009).
18. Adam, V. *et al.* Rational Control of Off-State Heterogeneity in a Photoswitchable Fluorescent Protein Provides Switching Contrast Enhancement. *ChemPhysChem* **23**, e202200192 (2022).
19. Bourges, A. C. *et al.* Quantitative determination of the full switching cycle of photochromic fluorescent proteins. *Chem. Commun.* **59**, 8810–8813 (2023).
20. Ringemann, C. *et al.* Enhancing Fluorescence Brightness: Effect of Reverse Intersystem Crossing Studied by Fluorescence Fluctuation Spectroscopy. *ChemPhysChem* **9**, 612–624 (2008).
21. Duan, C. *et al.* Structural Evidence for a Two-Regime Photobleaching Mechanism in a Reversibly Switchable Fluorescent Protein. *J. Am. Chem. Soc.* **135**, 15841–15850 (2013).
22. Roy, A., Field, M. J., Adam, V. & Bourgeois, D. The Nature of Transient Dark States in a Photoactivatable Fluorescent Protein. *J. Am. Chem. Soc.* **133**, 18586–18589 (2011).
23. Ha, T. & Tinnefeld, P. Photophysics of Fluorescent Probes for Single-Molecule Biophysics and Super-Resolution Imaging. *Annual Review of Physical Chemistry* vol. 63 595–617 (2012).
24. Schuster, J., Cichos, F. & Von Borczyskowski, C. Blinking of single molecules in various environments. *Opt. Spectrosc.* **98**, 712–717 (2005).
25. Sinnecker, D., Voigt, P., Hellwig, N. & Schaefer, M. Reversible Photobleaching of Enhanced Green Fluorescent Proteins. *Biochemistry* **44**, 7085–7094 (2005).

26. Berardozzi, R., Adam, V., Martins, A. & Bourgeois, D. Arginine 66 Controls Dark-State Formation in Green-to-Red Photoconvertible Fluorescent Proteins. *J. Am. Chem. Soc.* **138**, 558–565 (2016).
27. Chmyrov, A. *et al.* Nanoscopy with more than 100,000 'doughnuts'. *Nat. Methods* **10**, 737–740 (2013).
28. Grotjohann, T. *et al.* Diffraction-unlimited all-optical imaging and writing with a photochromic GFP. *Nature* **478**, 204–208 (2011).
29. Ando, R., Flors, C., Mizuno, H., Hofkens, J. & Miyawaki, A. Highlighted Generation of Fluorescence Signals Using Simultaneous Two-Color Irradiation on Dronpa Mutants. *Biophys. J.* **92**, L97–L99 (2007).
30. Duwé, S. *et al.* Expression-Enhanced Fluorescent Proteins Based on Enhanced Green Fluorescent Protein for Super-resolution Microscopy. *ACS Nano* **9**, 9528–9541 (2015).
31. Qian, Y., Celiker, O. T., Wang, Z., Guner-Ataman, B. & Boyden, E. S. Temporally multiplexed imaging of dynamic signaling networks in living cells. *Cell* **186**, 5656-5672.e21 (2023).
32. Chouket, R. *et al.* Extra kinetic dimensions for label discrimination. *Nat. Commun.* **13**, 1482 (2022).
33. Valenta, H. *et al.* Separation of spectrally overlapping fluorophores using intra-exposure excitation modulation. *Biophys. Rep.* **1**, 100026 (2021).
34. Valenta, H. *et al.* Per-pixel unmixing of spectrally overlapping fluorophores using intra-exposure excitation modulation. *Talanta* **269**, 125397 (2024).
35. De Beer, D., Stoodley, P., Roe, F. & Lewandowski, Z. Effects of biofilm structures on oxygen distribution and mass transport. *Biotechnol. Bioeng.* **43**, 1131–1138 (1994).
36. Stewart, P. S. Diffusion in biofilms. *J. Bacteriol.* **185**, 1485–1491 (2003).
37. Hepworth, S. J., Leach, M. O. & Doran, S. J. Dynamics of polymerization in polyacrylamide gel (PAG) dosimeters: (II) modelling oxygen diffusion. *Phys. Med. Biol.* **44**, 1875–1884 (1999).
38. Ju, L.-K. & Ho, C. S. The measurement of oxygen diffusion coefficients in polymeric solutions. *Chem. Eng. Sci.* **41**, 579–589 (1986).
39. Marek, P., Velasco-Veléz, J. J., Doll, T. & Sadowski, G. Compensation for the influence of temperature and humidity on oxygen diffusion in a reactive polymer matrix. *J. Sens. Sens. Syst.* **3**, 291–303 (2014).
40. Serebrovskaya, E. O. *et al.* Light-induced blockage of cell division with a chromatin-targeted phototoxic fluorescent protein. *Biochem. J.* **435**, 65–71 (2011).
41. Berberan-Santos, M. N. & Martinho, J. M. G. The integration of kinetic rate equations by matrix methods. *J. Chem. Educ.* **67**, 375 (1990).
